# Psychometric validation of research domain criteria, general health, and clinical-psychosocial parts of the minimum dataset of the German Center for Mental Health—informing a Delphi process

**DOI:** 10.1007/s00406-026-02315-w

**Published:** 2026-08-22

**Authors:** Bernd R. Förstner, Sarah Jane Böttger, Timm Seegert, André Kerber, Yannik Terhorst, Andreas Heinz, Isabel Dziobek, Babett Voigt, Silvia Schneider, Nils Opel, Martin Walter, Urs Braun, Andreas Meyer-Lindenberg, Nikolaos Koutsouleris, Peter Falkai, Norbert Schmitz, Andreas J. Fallgatter, Ulrike Sünkel, Silke Lipinski, Michael A. Rapp, Mira Tschorn, MDS Expert Group List, Vera Birgel, Alice Braun, Christoph Correll, Caroline Cohrdes, Sonja Entringer, Paul Gellert, Melissa Gül Halil, Sven Hädel, Christine Heim, Christine Knaevelsrud, Julia Kraft, Stephan Ripke, Matthias Rose, Henrik Walter, Julia Brailovskaia, Britta Bredenbröcker, Robert Kumsta, Maike Luhmann, Jürgen Margraf, Friederike Räder, Svenja Schaumburg, Sabine Seehagen, Ilona Croy, Katja Lind, Rafael Mikolajczyk, Fabian Rottstädt, Lavinia-Alexandra Steinmann, Stefanie Bauer, Sabine C. Herpertz, Philipp Kling, Katharina Kubera, Bernd Lenz, Ulrich Reininghaus, Christian Rauschenberg, Emanuel Schwarz, Heike Tost, Maximilian Wilhelm, Katja Bertsch, Thomas Ehring, Peter Henningsen, Stefan Leucht, Isabel Maurus, Frank Padberg, Anne Ruef, Gerd Eschweiler, Vanessa Nieratschker, Andreas Stengel, Stephan Zipfel, Myriam Bea, Rüdiger Hannig, Franz-Josef Wagner, Niall Boyce, David Cicero, Bruce Cuthbert, Ophélia Godin, Ute Habel, Marion Leboyer, Wolfgang Lutz, Isabel Maurus, Marcus Mund, Jenni Pacheco, Alizé Rogge, Steven Roose, Marie-Hélène Soto

**Affiliations:** 1 Social and Preventive Medicine, Department of Sports and Health Sciences, University of Potsdam, Am Mühlenberg 9, 14476 Potsdam, Germany; 2 German Center for Mental Health (DZPG), Partner Site Berlin/Potsdam, Potsdam, Germany; 3 Division of Clinical Psychosocial Intervention, Freie Universität Berlin, Berlin, Germany; 4 Department of Psychology, LMU Munich, Munich, Germany; 5 German Center for Mental Health (DZPG), Partner Site Munich-Augsburg, Munich, Germany; 6 German Center for Mental Health (DZPG), Central Trialogical Board, Berlin, Germany; 7 German Center for Mental Health (DZPG), Partner Site Bochum/Marburg, Bochum, Germany; 8 German Center for Mental Health (DZPG), Partner Site Halle/Jena/Magdeburg, Jena, Germany; 9 German Center for Mental Health (DZPG), Partner Site Mannheim/Heidelberg/Ulm, Mannheim, Germany; 10 German Center for Mental Health (DZPG), Partner Site Tübingen, Tübingen, Germany; 11 Department of Psychiatry and Psychotherapy, University Hospital, LMU Munich, Munich, Germany; 12 Max Planck Institute of Psychiatry, Munich, Germany; 13 Department of Psychiatry and Psychotherapy, Jena University Hospital, Jena, Germany; 14 Department of Psychiatry and Psychotherapy, University of Tübingen, Tübingen, Germany; 15 Department of Psychiatry and Psychotherapy and CRC TR 265, TU Dresden, Dresden, Germany; 16 Humboldt-Universität Zu Berlin, Berlin, Germany; 17 Central Institute for Mental Health, Medical Faculty Mannheim, Heidelberg University, Heidelberg, Germany; 18 Mental Health Research and Treatment Center (FBZ), Ruhr University Bochum, Bochum, Germany; 19 Department of Psychiatry & Neuroscience, Campus Benjamin Franklin, Charité Universitätsmedizin Berlin, Berlin, Germany; 20 Department of Clinical Child and Adolescent Psychology, Ruhr-University Bochum, Bochum, Germany; 21 Population-Based Medicine, University of Tübingen, Tübingen, Germany; 22 Klinische Psychologie Sozialer Interaktion, Institut Für Psychologie, Humboldt-Universität Zu Berlin, Berlin, Germany

**Keywords:** Minimum Dataset, MDS, DZPG, German Center for Mental Health, MGCFA, Transdiagnostic

## Abstract

**Objective:**

This study evaluated the latent structure and conceptual validity of the Research Domain Criteria (RDoC), General Health, and Clinical/Psychosocial clusters of the German Center for Mental Health (DZPG) Minimum Data Set (MDS). It aimed to determine whether these clusters represent interpretable constructs and demonstrate cross-group comparability across clinical and non-clinical populations, providing a data-driven basis for item selection within a Delphi consensus process.

**Methods:**

Two subsamples were analyzed: a representative sample (*N* = 2,287) and a clinic-recruited sample (*N* = 322) from six DZPG sites. Multi-group confirmatory factor analyses tested the latent structure and assessed configural, metric, and scalar measurement invariance between non-clinical vs. clinical/help-seeking groups.

**Results:**

All three MDS clusters demonstrated excellent baseline model fit (RDoC: CFI = .990, RMSEA = .037; General Health: CFI = .993, RMSEA = .037; Clinical/Psychosocial factors: CFI = .992, RMSEA = .026). Analyses confirmed configural and scalar invariance across all clusters but indicated non-invariance at the metric level for the General Health cluster.

**Conclusions:**

The results provide initial evidence for the structural validity and cross-group comparability of the RDoC and Clinical/Psychosocial clusters, while indicating limitations regarding metric non-invariance within the General Health cluster. They informed a Delphi consensus process and support the MDS as a scalable framework for diverse populations, particularly relevant for the DZPG’s effort to harmonize mental health data across institutions and contexts.

**Supplementary Information:**

The online version contains supplementary material available at https://doi.org/10.1007/s00406-026-02315-w.

## Introduction

The development of Minimum Data Sets (MDS) has gained increasing attention in various fields of health research, aiming to create standardized, efficient, and widely applicable assessment tools (e.g. [1]). MDS approaches have been successfully implemented in areas such as psychiatric emergency services [2], mental health outcomes [3], and health outcomes measurement in psychiatric care [4]. Serving as practical solutions, MDS approaches balance comprehensive data collection with feasibility in both clinical and research settings, enhancing data-driven decision-making and ultimately improving patient care. Moreover, standardized datasets enable the pooling and comparison of data across studies and sites, thereby facilitating large-scale analyses and the generation of generalizable insights.

Accurate and comprehensive psychometric measurement is fundamental to advancing research and clinical practice in mental health. Over the past decades, various theoretical and methodological frameworks have been established to capture the complexity of psychopathology or mental health. Relevant frameworks include models of transdiagnostic and/or dimensional models of mental functioning, such as the Hierarchical Taxonomy of Psychopathology (HiTOP) [5], the Research Domain Criteria (RDoC) [6] that organizes mental functions across domains and units of analysis, as well as broad assessments of General Health [7] or well-being [8]. Together, these frameworks cover complementary aspects of mental health, including dimensional psychopathology (HiTOP), transdiagnostic mental functioning (RDoC), and subjective health and functioning (General Health). Their integration therefore provides a comprehensive yet scalable foundation for mental health research. However, despite significant progress, the field continues to face challenges related to the standardization, feasibility, and cross-context applicability of psychometric assessments [9].

In response to these challenges, the development of a MDS in mental health provides a systematic approach to harmonizing psychometric assessment while maintaining efficiency in practical settings. A well-structured MDS should balance brevity and comprehensiveness to ensure clinical utility and psychometric robustness, while ensuring that the most essential aspects of the targeted constructs are adequately captured for both patient populations, healthy controls and representative epidemiological samples. The present initiative is embedded within the framework of the German Center for Mental Health (DZPG) [10] and builds upon RDoC, HiTOP, and validated constructs of General Health as well as Clinical and Psychosocial assessments including risk and protective factors. The DZPG is a nationwide research consortium funded by the German Federal Ministry of Research, Technology and Space (BMFTR). Its overarching goal is to advance translational mental health research and facilitate the implementation of scientific findings into routine care. Within this framework, the present MDS initiative was developed to establish a harmonized assessment strategy across sites and studies.

Therefore, the DZPG MDS expert workgroup initiated a Delphi consensus process to develop this harmonized core assessment in response to the growing need for standardized mental health data collection. This initiative involved expert researchers from all DZPG sites alongside representatives from (inter-)national centers of excellence, including the National Institute for Mental Health (NIMH). The primary objective was to define a concise, universally applicable MDS that provides a standardized baseline across studies while remaining modularly extendable. To achieve international comparability and enable seamless integration with large-scale cohorts, construct selection was anchored in the aforementioned internationally recognized transdiagnostic dimensional frameworks, integrating essential information across a broad range of mental health domains – such as sociodemographic background, clinical history and early adversity – while embedding selected items from the Patient Reported Outcome Measurement Information System (PROMIS) [11] to capture standardized patient-reported outcomes. The battery also incorporates further general health indicators (e.g. EQ-5D-5L (EuroQol) [12]) alongside validated measures of risk and protective factors, including variables like perceived stress, resilience, and experiences of social discrimination. Consequently, the MDS synthesizes dimensional frameworks with empirically validated indicators of mental health functioning, and well-being establishing a scalable foundation for harmonized data collection across diverse clinical and research settings.

A key component of the Delphi consensus process was testing the MDS Core battery with diverse participant groups to ensure its applicability, evaluate latent structure validity, and implement a data-driven item reduction strategy. This validation process is critical for ensuring the effectiveness and adaptability of the MDS for a wide range of populations. Thereby enhancing its potential for broad application in mental health research and practice. To ensure a harmonized MDS can be validly applied across diverse populations, its underlying constructs must be measured equivalently. Measurement invariance guarantees that observed variations between clinical and non-clinical cohorts represent true differences in the latent traits, rather than artifacts of item interpretation or performance. Demonstrating this invariance is thus a fundamental prerequisite for conducting pooled analyses, making valid cross-group comparisons, and establishing a unified assessment framework.

To this end, this study aimed to map the latent structure of three key MDS components and to evaluate their measurement invariance at the configural, metric, and scalar levels. Using a combined dataset of general population, clinical and help-seeking individuals, we hypothesized that the core three clusters (RDoC, General Health, and Clinical and Psychosocial Factors) would demonstrate robust invariance across clinical/help-seeking and non-clinical groups in those clusters. This psychometric equivalence would confirm that the latent constructs remain comparable and interpretable across both clinical/help-seeking and non-clinical groups. Beyond evaluating construct validity, these psychometric analyses were conducted to inform subsequent Delphi consensus decisions regarding item retention and reduction.

## Methods

### Participants and procedures

Participants were recruited as part of the MDS Valid study, which included two subsamples. The first subsample was drawn from a representative community panel recruited by a market research institute. These Participants were selected according to predefined quotas for gender (50:50 distribution), age groups (18–29, 30–39, 40–49, 50–59, and 60–69 years; except 70 +), and self-reported general health status [7], based on percentage distributions provided by the RKI from the German Socio-Economic Panel (SOEP) [13]. Study invitations were issued based on eligibility and random selection within the panel's quality and engagement protocols. The second subsample comprised participants recruited through six participating clinical DZPG sites: Mannheim/Heidelberg/Ulm, München/Augsburg, Tübingen, Marburg/Bochum, Halle/Jena/ Magdeburg, and Berlin/Potsdam.

Inclusion criteria required either participation in the representative community panel or current treatment at one of the DZPG sites, as well as informed consent to anonymized study participation and procedures. Participation in the representative community panel required adherence to the panel’s quality and engagement protocols, with study invitations contingent upon demographic eligibility and random selection. Panel participants received monetary compensation as an incentive for their involvement. No exclusion criteria were applied to either subsample. As a result, individuals with acute mental or significant medical conditions were not systematically excluded.

Data collection was conducted from February 23 to March 29, 2024, for the market research panel sample, and from January 30 to April 29, 2024, for the clinic-recruited sample. Ethical approval for the study was granted by the Ethics Committee of the University of Potsdam (Nr. 6/2016 Addendum MDS-Valid (1. 62/2019)), and the study adhered to the latest version of the Declaration of Helsinki and the German Data Protection Act (BDSG) in accordance with the GDPR. All participants were informed about the aims and procedures of the study prior to participation.

After data collection, participants from both subsamples were categorized into two groups based on self-reported mental health status: (1) healthy controls and (2) individuals with a self-reported mental health diagnosis and/or those initiating contact for mental health treatment. Sociodemographic characteristics are reported in the Results section (see Table 2). The questionnaire was available for completion via PC, laptop, or mobile device to ensure accessibility across platforms. Informed consent was obtained electronically via confirmation on the survey platform. Participation in the study was fully anonymized.

### Measurements

**Fig. 1:**
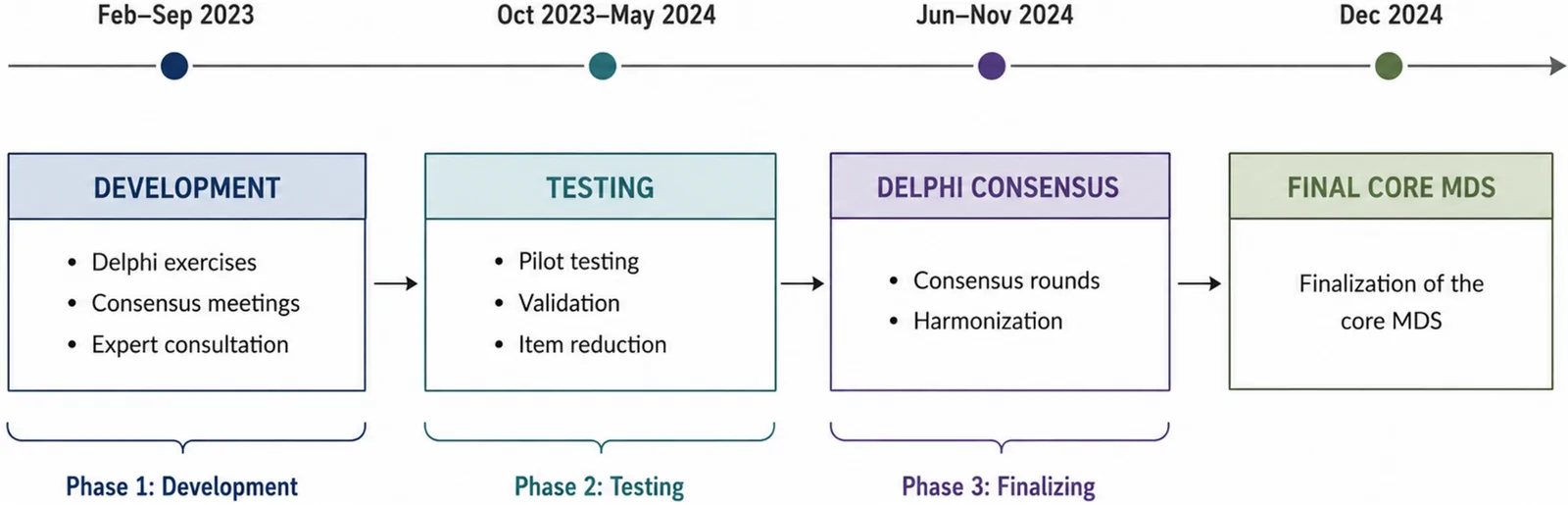
*MDS Delphi process timeline.* Reproduced from Tschorn et al. (2025) [14], available on OSF.io under a Creative Commons Attribution 4.0 International License

Data collection was based on a comprehensive psychometric questionnaire battery that was initially developed through multiple Delphi consensus rounds involving DZPG experts and international collaborators (see also osf.io/ec9hs/). These initial Delphi rounds resulted in a preliminary core battery for the Minimum Dataset (MDS), which was subsequently subjected to psychometric validation and measurement invariance analyses in the present study. In addition to evaluating the factorial structure and cross-group comparability of the instrument, the validation procedure also aimed to identify opportunities for item reduction in line with the intended administration time of the MDS. The findings from this validation study subsequently informed further Delphi consensus rounds and harmonization procedures that led to the final MDS core battery (see also Fig. 1).

The self-report instruments were spread across sociodemographic information and four clusters measured: Research Domain Criteria (RDoC), Hierarchical Taxonomy of Psychopathology (HiTOP), General Health (incl. PROMIS), and Clinical and Psychosocial Factors. In line with the conceptual rationale outlined above, the RDoC variables were retained as a separate cluster to preserve the integrity of this transdiagnostic framework. Based on prior Delphi consensus decisions, the PROMIS and HiTOP constructs were not allocated to independent clusters. For PROMIS, the Delphi consensus resulted in the selection of four broadly formulated, non–disease-specific questions, making it more appropriate to combine them with other variables assessing general health in a unified General Health cluster. For HiTOP, the hierarchical dimensional structure of psychopathology required a dedicated analysis based on additional data [15] that explicitly accounted for this organization, which is reported elsewhere. Nonetheless, some of the Clinical and Psychosocial cluster items can be used as linkage variables to the HiTOP internalizing spectrum, alongside other risk and protective factors. More information on this Delphi consensus process as well as all final items, respective codebook and source information for the MDS can be accessed at Open Science Framework (OSF; osf.io/ec9hs/).

Notably, conceptual and empirical overlap between clusters at the item level was intentionally maintained to allow for the capture of multiple domains with shared indicators. This approach reflects the transdiagnostic and multidimensional nature of mental health constructs and enhances the integrative value of the MDS across theoretical frameworks. The following summarizes the measured structures within the clusters. A detailed list of the three clusters, their underlying domain factor structure and the respective items used can be found in Table 1. Items were rated using Likert or visual analogue scales (VAS) or bubble ratings [16, 17] as response formats. To enhance interpretability, the first variable of each factor was recoded, if necessary, to ensure a positive direction, meaning higher values indicate a more plausible factor expression. All items were presented in the German language across different the aforementioned platforms.

**Table 1:** Specifications on Domains, Items, Assessments

Domain	Assessment, orig. Item no	Item	*Ref*	*Mean* (*SD*)	Mdn	% Miss
RDoC						
PVS	PANAS active PANAS determined [R] PANAS excited PANAS interested [R]	*Active* *Determined* *Excited* *Interested*	[30]	3.19 (1.01) 3.14 (1.09) 3.01 (1.05) 3.47 (0.94)	3 3 3 4	0.0 0.0 0.0 0.0
	POMS full of pep^a^ [R]	*Full of pep*	[48]; german sf 35 items, [25]	2.87 (1.43)	3	0.0
NVS	SUDS raw score	*Think of the worst anxiety you have ever experienced or can imagine experiencing and assign to this the number 100. Now think of the state of being absolutely calm and call this zero. Now you have a scale. On this scale how do you rate yourself at this moment?*	[62]	31.12 (27.59)	20	0.0
	CCSM, 6^d^ [R] CCSM, 7 [R] CCSM, 8 _modified_ [R]	*Feeling nervous, anxious, frightened, worried, or on edge?* *Feeling panic or being frightened?* *Avoiding situations/things/places/activities that make you anxious (e.g., open spaces, to travel on buses, subways, or trains, feeling uneasy in crowds)?*	[26]	1.26 (1.17) 0.63 (0.96) 0.76 (1.09)	1 0 0	0.0 0.0 0.0
	PID-5, 64	*I can’t stand being left alone, even for a few hours*	[47]	0.35 (0.69)	0	0.0
	PANAS ashamed PANAS irritable	*Ashamed* *Irritable*	[30]	1.58 (0.95) 2.27 (1.06)	1 2	0.0 0.0
CS	CCSM, 15 [R]	*Problems with memory (e.g., learning new information) or with location (e.g., finding your way home)?*	[26]	3.33 (0.98)	4	0.0
	Memory based on BRIEF, altered [R] Flexibility based on BRIEF, altered Planning based on BRIEF, altered [R]	*[I have difficulties remembering information or things or keeping tasks in mind]*^*d*^ *[When solving tasks, I have difficulties finding new solutions when I get stuck]*^*d*^ *[I have difficulties planning or performing complex tasks or activities that require multiple action steps]*^*d*^	-	1.64 (0.65) 1.50 (0.62) 1.43 (0.62)	2 1 1	0.0 0.0 0.0
SP	CCSM, 1 modified [R] CCSM, 3 [R]	*Little interest or pleasure in doing things in the company of others?* *Feeling more irritated, grouchy, or angry than usual*	[26]	1.33 (1.16) 1.32 (1.05)	1 1	0.0 0.0
	WHO-DAS 2.0, 11	*In the past 30 days, how much difficulty did you have in: maintaining a friendship?*	[58]	3.0 (1.18)	3	0.0
	LPSF-BF, 7	*I often have difficulty understanding the thoughts and feelings of others*	[40, 55]	1.82 (0.78)	2	0.0
	Social exclusion based on social discrimination [R]	*Overall, how socially excluded or isolated have you felt in the last 30 days?*	SOEP adapted [13]	23.39 (27.75)	11	0.1
	LHA, 4 based on LHA, modified LHA, 4a new follow-up item	*Have you ever physically assaulted another person with the intention to cause harm?* *Has this happened in the last 6 months?*	[32]; own german transl	1.41 (0.87) 1.93 (0.25)	1 2	0.0 76.7
ARS	POMS worn-out [R] POMS fatigued [R] POMS exhausted [R]	*Worn-out* *Fatigued* *Exhausted*	[48]; german sf, 35 items, [25]	2.60 (1.82) 2.92 (1.70) 2.16 (1.77)	3 3 2	0.0 0.0 0.0
	CCSM, 14	*Problems with sleep that affected your sleep quality over all?*	[26]	2.55 (1.20)	3	0.0
SMS	AES, 2 [R] AES, 3 [R] AES, 10 AES, 16 AES, 17 [R]	*I get things done during the day* *Getting things started on my own is important to me* *Someone has to tell me what to do each day* *Getting things done during the day is important to me* *I have initiative*	[45, 46]; german vers. [44]	3.22 (0.75) 3.08 (0.79) 1.33 (0.68) 2.80 (0.87) 3.12 (0.81)	3 3 1 3 3	0.0 0.0 0.0 0.0 0.0
General health						
MeH	EPO-1 [R], VAS	*How well do you feel you are getting along emotionally and psychologically?*	[36]	76.14 (24.45)	85	0.0
	PROMIS, GMH-2, global 04 PROMIS, GMH-2, global 05	*In general, how would you rate your mental health, including your mood and your ability to think?* *In general, how would you rate your satisfaction with your social activities and relationships?*	[11]	3.34 (1.08) 3.22 (1.05)	3 3	0.0 0.0
	EQ-5D-5L, 4^a^	*Anxiety/Depression*	[12]	1.71 (0.78)	2	0.0
PhF	MEHM, self-perceived health [R]	*How is your health in general?*	[7]	3.77 (0.83)	4	0.0
	PROMIS, GPH-2, global 03 PROMIS, GPH-2, global 06^a^	*In general, how would you rate your physical health?* *To what extent are you able to carry out your everyday physical activities such as walking, climbing stairs, carrying groceries, or moving a chair?*	[11]	3.20 (0.90) 4.61 (0.72)	3 5	0.0 0.0
	EQ-5D-5L, 1^b^	*Mobility*	[12]	1.30 (0.66)	1	0.0
FHL	MEHM, CC modified MEHM, GALI, 1 modified [R] MEHM, GALI, 2 modified [R]	*Do you suffer from any long-standing illness or condition (health problem)? This refers to illnesses or health conditions that persist or are expected to persist for a duration of at least six months* *Have you been limited in activities people usually do because of health problem?* *For how long have you been limited?*	[7]	1.42 (0.49) 1.32 (0.54) 0.56 (0.88)	1 1 0	0.0 0.0 0.0
	EQ-5D-5L, 1^b^ EQ-5D-5L, 5^b^	*Mobility* *Pain/discomfort*	[12]	1.30 (0.66) 1.72 (0.96)	1 1	0.0 0.0
QoL	EQ-5D-5L, 2 EQ-5D-5L, 3 [R] EQ-5D-5L, 5^b^ EQ-5D-5L, VAS	*Self-Care* *Usual activities* *Pain/discomfort* *Health state, today*	[12]	1.08 (0.36) 4.61 (0.79) 1.72 (0.96) 75.46 (18.59)	1 5 1 80	0.0 0.0 0.0 0.0
SWB	Worthwhileness, VAS modified Happiness, VAS modified [R] Life satisfaction, VAS modified Life satisfaction, bubble rating PMH, bubble rating	*Overall, to what extent do you feel that the things you do in your life are worthwhile?* (eudemonic well-being) *Overall, how happy did you feel nowadays?* (affective-evaluative component of hedonic well-being) *Overall, how satisfied are you with your life nowadays?* (cognitive-evaluative component of hedonic well-being) *Satisfaction—excellent life conditions—almost perfect—get the important things—would change almost nothing* *Carefree—pleasure of life—life satisfaction—confidence—management of difficulties—joy—calm and balance*	[28, 33, 45, 57]	71.70 (24.47) 68.77 (25.09) 68.87 (24.12) 1.74 (0.85) 1.71 (0.83)	80 75 75 2 2	0.04 0.04 0.04 0.04 0.08
Clinical and Psychosocial factors						
CTrauma	CTS, 1^a^ [R] CTS, 2^a^ [R] CTS, 3^a^ [R] CTS, 4^a^ [R] CTS, 5^a^ [R]	*I felt loved* *There was someone to take me to the doctor when I needed it* *People in my family hit me so hard, it left me with bruises or marks* *I felt that somebody in my family hated me* *Somebody molested me*	[37]	2.90 (1.07) 0.47 (0.91) 0.62 (1.06) 0.20 (0.62) 3.06 (1.13)	3 0 0 0 3	0.0 0.0 0.0 0.0 0.0
Trauma Exp	LEC-5 based on LEC-5 items 6 and 8 [R] LEC-5, 10 [R] LEC-5 based on LEC-5 items 1, 2, 4 and 17	*Physical or sexual assault* *Combat or exposure to a war-zone* *Natural disaster, serious accident, fire or explosion or any other stressful event or experience*	[60] (National Center for PTSD); ger. vers. [34]	0.60 (0.81) 0.18 (0.47) 0.41 (0.64)	0 0 0	0.0 0.0 0.0
Trauma Seq	PC-PTSD-5, 0 PC-PTSD-5, 1 PC-PTSD-5, 2 [R] PC-PTSD-5, 3 [R] PC-PTSD-5, 4 PC-PTSD-5, 5	*Sometimes things happen to people that are unusually or especially frightening, horrible, or traumatic. Have you ever experienced this kind of event?* *Had nightmares about the event(s) or thought about the event(s) when you did not want to?* *Tried hard not to think about the event(s) or went out of your way to avoid situations that reminded you of the event(s)?* *Been constantly on guard, watchful, or easily startled?* *Felt numb or detached from people, activities, or your surroundings?* *Felt guilty or unable to stop blaming yourself or others for the event(s) or any problems the event(s) may have caused?*	[53]; ger. vers. [54]	1.70 (0.46) 0.50 (0.80) 0.49 (0.80) 0.51 (0.83) 0.48 (0.79) 0.51 (0.83)	2 0 0 0 0 0	0.0 0.04 0.04 0.04 0.04 0.04
Depr	PHQ-4, 1 [R] PHQ-4, 2 [R]	*Little interest or pleasure in doing things* *Feeling down, depressed, or hopeless*	[56]	0.75 (0.83) 0.59 (0.84)	1 0	0.0 0.0
	DASS-21, 3 [R] DASS-21, 5 DASS-21, 10 DASS-21, 13 DASS-21, 16 DASS-21, 17 [R] DASS-21, 21 DASS-21, depr, bubble rating	*I couldn't seem to experience any positive feeling at all* *I found it difficult to work up the initiative to do things* *I felt that I had nothing to look forward to* *I felt down-hearted and blue* *I was unable to become enthusiastic about anything* *I felt I wasn't worth much as a person* *I felt that life was meaningless* *Sad—no initiative—joyless—depressed—no interest—worthless—meaningless*	[43]; ger. vers. [52]	0.45 (0.76) 0.88 (0.90) 0.57 (0.87) 0.78 (0.91) 0.57 (0.85) 0.58 (0.91) 0.40 (0.79) 0.78 (0.86)	0 1 0 1 0 0 0 1	0.0 0.0 0.0 0.0 0.04 0.04 0.0 0.08
	CCSM, 1^a^	*Little interest or pleasure in doing things?*	[26]	1.28 (1.11)	1	0.0
Anx	PHQ-4, 3 [R] PHQ-4, 4 [R]	*Feeling nervous, anxious or on edge* *Not being able to stop or control worrying*	[56]	0.61 (0.81) 0.55 (0.84)	0 0	0.0 0.0
	DASS-21, 2 DASS-21, 4 DASS-21, 7 DASS-21, 9 DASS-21, 15 DASS-21, 19 DASS-21, 20 DASS-21, anx, bubble rating	*I was aware of dryness of my mouth* *I experienced breathing difficulty (e.g., excessively rapid breathing, breathlessness in the absence of physical exertion)* *I experienced trembling (e.g. in the hands)* *I was worried about situations in which I might panic and make a fool of myself* *I felt I was close to panic* *I was aware of the action of my heart in the absence of physical exertion (e.g., sense of heart rate increase, heart missing a beat)* *I felt scared without any good reason* *Dryness of mouth – breathlessness – tremble – worries – panic – beating of heart – groundless scared*	[43]; ger. vers. [52]	0.53 (0.75) 0.31 (0.65) 0.30 (0.65) 0.44 (0.79) 0.29 (0.65) 0.50 (0.79) 0.38 (0.74) 0.46 (0.70)	0 0 0 0 0 0 0 0	0.0 0.0 0.0 0.0 0.0 0.0 0.0 0.34
	CCSM, 6^b^ [R]	*Feeling nervous, anxious, frightened, worried, or on edge?*	[26]	1.26 (1.17)	1	0.04
Stress	DASS-21, 1 [R] DASS-21, 6 DASS-21, 8 [R] DASS-21, 11 DASS-21, 12 [R] DASS-21, 14 DASS-21, 18 DASS-21, stress, bubble rating	*I found it hard to wind down* *I tended to over-react to situations* *I felt that I was using a lot of nervous energy* *I found myself getting agitated* *I found it difficult to relax* *I was intolerant of anything that kept me from getting on with what I was doing* *I felt that I was rather touchy* *Restless—irritated—tense—agitated—overexcited—thin-skinned—sensitive*	[43]; ger. vers. [52]	0.54 (0.78) 0.62 (0.81) 0.77 (0.89) 0.75 (0.84) 0.87 (0.93) 0.45 (0.74) 0.69 (0.88) 0.96 (0.77)	0 0 0 1 1 0 0 1	0.0 0.0 0.0 0.0 0.04 0.0 0.0 0.11
Eat	SNAQ, 3	*Over the last month: Did you experience a decreased appetite?*	[42]	1.85 (0.36)	2	0.08
	EAT-26, 23 [R]	*Engage in dieting behavior*	[35]; ger. vers. [49]	1.78 (1.43)	2	0.0
Som	CCSM, 9 CCSM, 10 [R]	*Unexplained aches and pains (e.g., head, back, joints, abdomen, legs)?* *Feeling that your illnesses are not being taken seriously enough?*	[26]	1.11 (1.08) 0.76 (1.10)	1 0	0.0 0.04
SoC	SoC, 1 [R] SoC, 2	*Do you experience important areas of your life (i.e., work, freetime, family, *etc*.) to be uncontrollable, meaning that you cannot, or barely can, influence them?* *Do you experience these important areas of your life as unpredictable or inscrutable?*	[51]	1.10 (1.11) 1.00 (1.04)	1 1	0.0 0.0
Social	Loneliness, SI Direct frequency [R]	*How often do you feel lonely?*	[50]	2.32 (1.06)	2	0.0
	IPSM, 24^a^ IPSM, 30^a^ IPSM, 33^a^ IPSM, 36^a^	*If other people knew what I am really like, they would think less of me* *I worry about what others think of me* *I worry about hurting the feelings of other people* *I care about what people feel about me*	[29, 39]	1.71 (0.92) 2.30 (1.00) 2.39 (1.00) 2.41 (0.96)	1 2 2 2	0.0 0.0 0.0 0.0
	Social discrimination, modified^a^	*Discrimination means that a person is treated worse than other people for certain reasons without any factual justification How much have you felt discriminated against or experienced discrimination in the last 30 days?*	based on SOEP [13]	10.29 (19.76)	1	0.2
	OSSS-3, 2^a^	*How much interest and concern do people show in what you do?*	Based on KIGGS (RKI) [41]	3.27 (0.84)	3	0.0
	Social support, bubble rating^a^	*Understanding and security—person of trust—support on-demand—acquantances—friends*	[16, 17]	1.98 (0.79)	2	0.0
Suppr	ERQ, suppression, 2^a^ [R] ERQ, suppression, 6^a^	*I keep my emotions to myself* *I control my emotions by not expressing them*	[27, 38]; ger. vers. [24]	4.42 (1.58) 4.47 (1.60)	4 5	0.0 0.1
ReApp	ERQ, reappraisal, 7^a^ [R] ERQ, reappraisal, 8^a^	*When I want to feel more positive emotion, I change the way I’m thinking about the situation* *I control my emotions by changing the way I think about the situation I’m in*	[27, 38]; german vers. [24]	4.28 (1.51) 4.13 (1.49)	4 4	0.1 0.1
Resil	CD-RISC-2, 1 CD-RISC-2, 8	*I am able to adapt to change* *I tend to bounce back after illness or hardship*	[59]; ger. vers. [61]	2.83 (0.78) 2.92 (0.85)	3 3	79.85 79.85
	BRS, 1^c^ [R] BRS, 2^c^ BRS, 3^c^ BRS, 4^c^ BRS, 5^c^ BRS, 6^c^ [R]	*I tend to bounce back quickly after hard times* *I have a hard time making it through stressful events* *It does not take me long to recover from a stressful event* *It is hard for me to snap back when something bad happens* *I usually come through difficult times with little trouble* *I tend to take a long time to get over set-backs in my life*	[18]; ger. vers. [31]	3.55 (1.03) 2.44 (1.11) 3.33 (1.13) 2.66 (1.15) 3.50 (1.04) 2.54 (1.13)	4 2 4 3 4 2	12.16 12.16 12.16 12.16 12.16 12.16

Details on the response formats can be found in the codebook on OSF.io. *AES* Apathy Evaluation Scale, *Anx* Anxiety, *ARS* Arousal and Regulatory Systems, *BRIEF* Behavior Rating Inventory of Executive Function, *BRS* Brief Resilience Scale, *CCSM* DSM-5 Level 1 Cross-Cutting Symptom Measure, *CD-RISC-2* Connor-Davidson Resilience Scale-2 Item Short Form, *CS* Cognitive Systems, *CTrauma* Childhood Trauma, *CTS* Childhood Trauma Screener, *DASS-21* Depression Anxiety Stress Scales, *Depr* Depression, *EAT* Eating behavior, *EAT-26* Eating Attitudes Test, *EQ-5D-5L* European Quality of Life-5 Dimensions-5 Level Version, *EPO-1* Emotional and Psychological Outcome-1, *ERQ* Emotion Regulation Questionnaire, *FHL* Functional Health Limitations, *GMH* General Mental Health, *GPH* General Physical Health, *IPSM* Interpersonal Sensitivity Measure, *LEC-5* Life Events Checklist for DSM-5, *LHA* Life History of Aggression, *LPSF-BF* Level of Personality Functioning Scale-Brief Form, *MEHM, CC/GALI* Minimum European Health Module, Chronic Conditions/Global Activity Limitation Indicator, *OSSS-3* Oslo Social Support Scale, *PANAS* Positive and Negative Affect Scale, *PC-PTSD-5* Primary Care PTSD Screener, *MeH* Mental Health, *PhF* Physical Functioning, *PHQ-4* Patient Health Questionnaire-4, *PID-5* Personality Inventory for DSM-5, *POMS* Profile of Mood States, *PMH* Positive Mental Health, *PROMIS GMH-2/GPH-2* Patient Reported Outcomes Measurement and Information System Version 1.2, Global Mental Health/Global Physical Health, *PVS* Positive Valence Systems, QoL Quality of Life, *RDoC* Research Domain Criteria, *Reapp* Cognitive Reappraisal, Resil Resilience*, SI Direct*_*frequency*_ Direct Single-Item Frequency Measure, *SMS* Sensorimotor Systems, *SNAQ* Short Nutritional Assessment Questionnaire, *SoC* Sense of Control, *Social* Social Well-Being (functioning), *Som* Somatization, *SP* Social Processes, *SUDS* Subjective Units of Distress Scale, *Suppr* Suppression, *SWB* Subjective Well-Being, *Trauma Exp* Trauma experience, *Trauma Seq* Trauma Sequelae, *VAS* Visual Analogue Scale, *WHO-DAS 2.0* WHO Disability Assessment Schedule 2.0

Altered decisions from MDS results meeting after analysis: *Life satisfaction* was recommended, but *Happiness, VAS modified* was ultimately chosen. CTS-Items: decision to keep all items instead of using Items with [R]. PHQ-Items: decision to keep all items instead of using Items with [R]. *EAT-26, 23 was recommended, but both EAT-26, 23 and SNAQ, 3 were ultimately retained*. *CCSM, 10 was recommended, but both CCSM, 10 and CCSM, 9 were ultimately retained*

[R] Item was recommended for inclusion/selection as a top outcome measure in the consensus-based development of the core MDS

^1^Item was reassigned to a different domain during the analytical process

^2^Item was reassigned to two domains during the analytical process

^3^Item was tested only in the representative community sample

^4^English item version not available, as the items were newly generated

### Statistics

The construct validation phase of this study was undertaken to ensure the broad applicability of the measures across diverse clinical and non-clinical study populations, as well as to support the Delphi consensus process by providing empirical guidance on the predefined constructs and the necessary item reduction for the development of the MDS. For each cluster (RDoC, General Health, and Clinical and Psychosocial), standardized multi-group confirmatory factor analyses (MGCFA) were conducted to compare participants based on their mental health status after categorization into the aforementioned groups. Analyses were performed using the weighted least squares mean and variance adjusted (WLSMV) estimator to accommodate the predominantly categorical (exception: VAS scales) nature of the data. Detailed information on the specific MGCFA steps, criteria, and deviations from this specific procedure is provided in supplementary material S1.

The overall proportion of missing data in the dataset (including sociodemographic data) was low at 7.19%. Within the individual clusters, the missing data rates were as follows: RDoC: 1.58%, General Health: 0.01%, and Mental Health: 3.89% (mostly stemming from missing Brief Resilience Scale (BRS) [18] assessment in the DZPG sample). The missing data mechanism was assumed to be missing at random (MAR), with exceptions for BRS items (MNAR in the clinical sample) and items in the Clinical and Psychosocial cluster with filter-dependent missingness. For those items, missing codes (-3, -2) were retained to avoid excessive data loss and ensure model convergence. No imputation was applied; analyses used available data. To evaluate the robustness of this approach, sensitivity analyses comparing FIML and WLSMV estimators were conducted for the final cluster models, indicating stable factor structures and consistent substantive results, with only minor deviations in global model fit indices, likely attributable to the more appropriate use of WLSMV for ordinal data. The resulting variations in sample sizes across analyses are reported accordingly.

Exploratory data analysis and the Shapiro-Wilks tests indicated non-normal distributions for all indicator variables, supporting the choice of the WLSMV estimator. Multicollinearity was assessed within each cluster using established cutoff criteria [19], revealing elevated multicollinearity in several variables, particularly within the Clinical and Psychosocial cluster. These findings were considered during model adjustments to mitigate multicollinearity effects and were carefully considered for each clusters analysis as well as in informing the Delphi consensus process. All analyses were performed using R (version 4.4.0) and RStudio (version 2024.04.0 + 735 "Chocolate Cosmos") with the lavaan package (version 0.6–19) [20]. The reporting of this observational study follows the STROBE (Strengthening the Reporting of Observational Studies in Epidemiology) guidelines [21].

## Results

A total of 2,619 individuals were included in the full sample, comprising two subsamples: a representative community sample (*n* = 2,297), recruited via a market research institute, and a clinical-recruited sample drawn from the DZPG institutional network (*n* = 322). The age range of participants was from 18 to 70 years (M = 43.8, SD = 14.6). The representative community sample was slightly older on average (M = 44.9, SD = 14.5) than the clinic-recruited sample (M = 35.6, SD = 12.9). Across the total sample, 52.1% identified as female and 47.8% as male. The proportion of female participants was substantially higher in the clinic-recruited sample (67.4%) compared to the representative community sample (43.9%). Regarding mental health status, 61.0% of participants in the full sample were classified as belonging to a “healthy” group without prior self-reported diagnosis or treatment contact, whereas 39.0% reported a mental health diagnosis or contact with psychiatric or psychotherapeutic services. This proportion varied markedly across subsamples.

Among the total sample, 25.7% reported at least one mental health diagnosis, with the most frequently reported conditions being unipolar depressive disorder (18.4%) and anxiety disorders (9.7%). As expected, rates of self-reported diagnoses were substantially higher in the DZPG sample, with 71.4% reporting at least one diagnosis. Comorbidity, defined as the presence of multiple diagnoses, was reported by 14.8% of the total sample and nearly half (47.5%) of the DZPG participants. First-aid contact with mental health services was reported by 37.6% of the full sample, with a significantly higher proportion observed in the clinic-recruited subsample (82.3%) compared to the representative community sample (31.4%). A comprehensive description of the sample is available in Table 2.

**Table 2:** Sample characteristics

Variable	Total sample	Representative community sample	Clinic-recruited sample
Subjects, N	2619	2297	322
Age, y			
Mean age (± *SD*)	43.8 (14.6)	44.94 (14.5)	35.6 (12.9)
Range	18–70	18–70	18–70
Gender, (%)			
Female	1364 (52.1)	1149 (50.0)	215 (66.7)
Male	1251 (47.8)	1148 (50.0)	103 (32.0)
First-aid contact, (%)	986 (37.6)	721 (31.4)	265 (82.3)
Mental Health Status, (%)			
(1) Healthy group	1597 (61.0)	1544 (67.2)	53 (16.5)
(2) Self-rep. diagnosis/ helpseeking group	1022 (39.0)	753 (32.8)	269 (83.5)
Self-reported diagnoses, (%)*	672 (25.7)	424 (18.5)	230 (71.4)
Anxiety Disorders	254 (9.7)	174 (7.6)	80 (24.8)
Unipolar Depressive Disorder	483 (18.4)	315 (13.7)	168 (52.2)
Psychotic Disorder	45 (1.7)	23 (1.0)	22 (6.8)
Bipolar Disorder	37 (1.4)	20 (0.9)	17 (5.3)
Obsessive Compulsive Disorder	54 (2.1)	27 (1.2)	27 (8.4)
Post Traumatic Stress Disorder	132 (5.0)	77 (3.4)	55 (17.1)
Somatoform Disorder	32 (1.2)	23 (1.0)	9 (2.8)
Eating Disorder	83 (3.2)	42 (1.8)	41 (12.7)
Personality and/or Behavioral disorder	112 (4.3)	53 (2.3)	59 (18.3)
Developmental Disorder	12 (0.5)	5 (0.2)	7 (2.2)
Attention Deficit Hyperactivity Disorder	40 (1.5)	21 (0.9)	19 (5.9)
Autism Spectrum Disorder	23 (0.9)	12 (0.5)	11 (3.4)
Substance Use Disorder	40 (1.5)	24 (1.0)	16 (5.0)
Comorbidity, (%)*	387 (14.8)	234 (10.2)	153 (47.5)

*SD* Standard deviation, *y* years

Representative community sample surveyed by a market research institute. Clinic-recruited sample surveyed throughout the DZPG institutional network; 1 diverse person was excluded from the analysis

^*^Self-reported Diagnosis (Multiple Choice Option)

The following sections present the results of the MGCFAs for the three examined clusters: RDoC, General Health, and Clinical and Psychosocial Factors. Only the outcomes of the final optimized models are reported here. Details regarding intermediate models, model comparisons, and alternative specifications are available in the corresponding Supplementary Materials (S2-S4).

### RDoC cluster

To evaluate the factorial validity of the selected RDoC variables and its latent factors Positive Valence Systems (PVS), Negative Valence Systems (NVS), Cognitive Systems (CS), Social Processes (SP), Arousal and Regulatory Systems (ARS) and Sensorimotor Systems (SMS), a series of CFA and MGCFA models were conducted to examine the internal structure, subgroup model fit, and measurement invariance across individuals with and without mental health diagnoses. Table 3 summarizes the results of all CFA and MGCFA models tested for the RDoC cluster. The initial configural model (Model 1) showed suboptimal fit and was therefore refined by excluding low-performing items (R^2^ < 0.20) and applying theory-driven modifications (see also Supplement S2 for details). The final configural model (Model 4) and the separate CFAs for both groups (Models 2/3) demonstrated very good fit, indicating adequate configural invariance. Metric invariance was supported under standard but not strict criteria (ΔCFI = 0.009), while scalar invariance met both conventional and strict thresholds (ΔCFI = 0.001). The final multidimensional model demonstrated excellent fit and significantly outperformed both a unidimensional model and a model with orthogonal factors. A reduced “TOP3-Item” version (Model 10) of the model further improved model fit and parsimony and was used to test the variables which went back into the Delphi consensus.

**Table 3:** RDoC (MG)CFA Models from stepwise procedure

No	Model description	N (Sample Size)	CFI	TLI	RMSEA	SRMR	χ^2^(test (comp mod.)	ΔCFI
1	MGCFA Model	2604 (1593/1011)	0.929	0.925	0.064	0.072	-	-
2	CFA Model Healthy Group	1595	0.986	0.985	0.038	0.044	-	-
3	CFA Model Clinical Group	1019	0.988	0.987	0.040	0.042	-	-
4	MGCFA Configural Model	2614 (1595/1019)	0.985	0.983	0.038	0.042	-	-
5	MGCFA Metric Invariance Model	2614 (1595/1019)	0.977	0.975	0.048	0.051	χ^2^(692) = 196.83^***^(4)	0.009^a^
6	MGCFA Scalar Invariance Model	2614 (1595/1019)	0.976	0.975	0.047	0.052	χ^2^(714) = 101.29^***^(5)	0.001^b^
7	CFA Final RDoC Model	2614	0.990	0.989	0.037	0.039	-	-
8	CFA One-Factor Model	2614	0.961	0.957	0.072	0.076	χ^2^(350) = 2150.40^***^(7)	-
9	CFA Orthogonal Factors Model	2611	0.242	0.182	0.316	0.324	χ^2^(350) = 4182.60^***^(7)	-
10	TOP 3 Item Model	2612	0.996	0.995	0.033	0.032	-	-

*CFA* Confirmatory Factor Analysis, *CFI* Comparative Fit Index, *Comp. mod.* compared model, *MGCFA* Multigroup Confirmatory Factor Analysis, *TFI* Tucker–Lewis Fit Index, *TLI* Tucker–Lewis Index, *RDoC* Research Domain Criteria, *RMSEA* Root Mean Square Error of Approximation, *SRMR* Standardized Root Mean Square Residual

All reported fit indices are based on robust estimators

^*^p < .05

^**^p < .01

^***^p < .001 (two-tailed)

ΔCFI values reflect comparisons to previous model steps as indicated in parentheses

^a^ΔCFI < .01 (acceptable threshold; Cheung & Rensvold, 2002)

^b^ΔCFI < .002 (strict criterion; Meade et al., 2008)

**Fig. 2:**
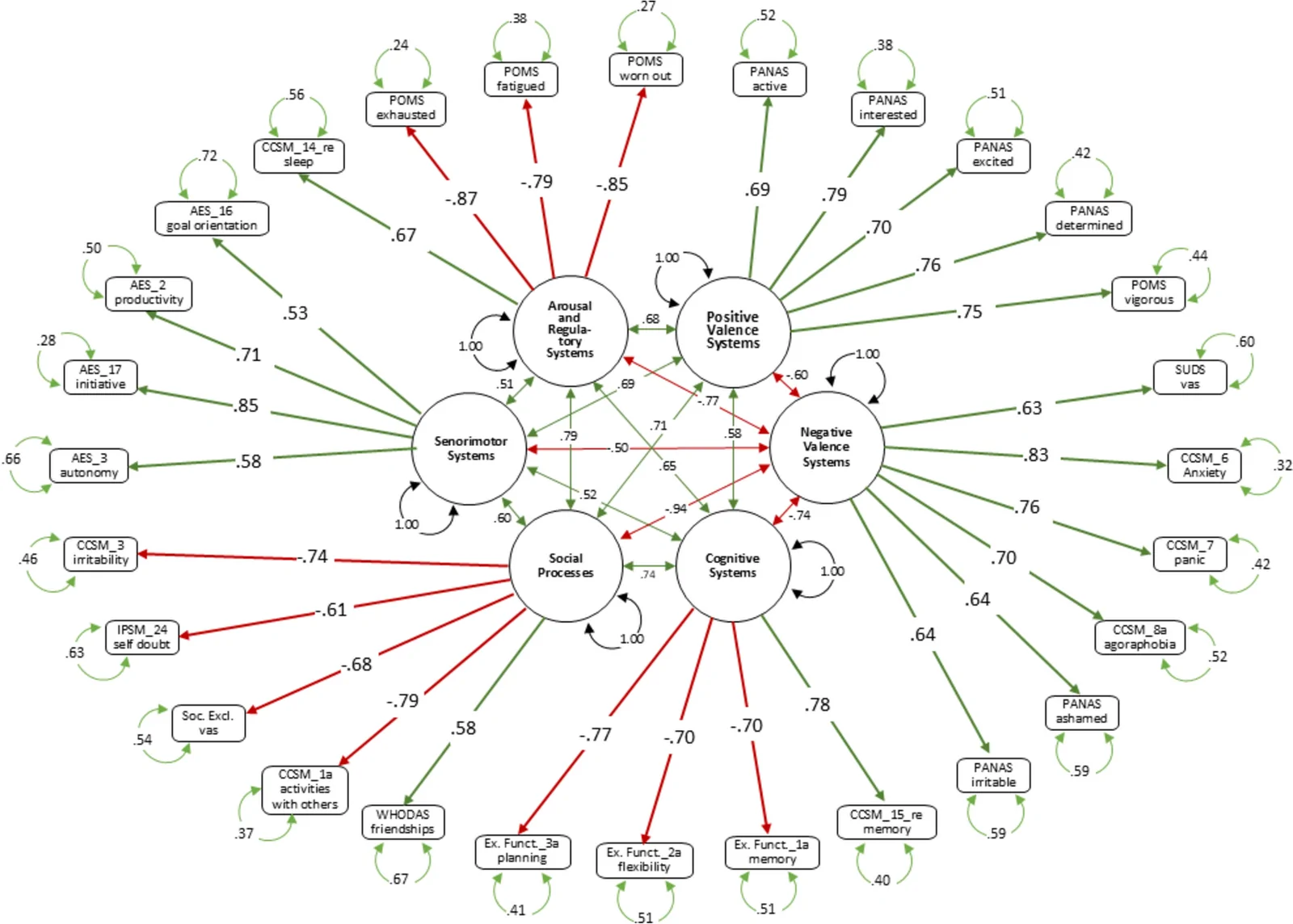
*Factorial loadings of the final model on the six RDoC domains.* **Note.** Standardized factor loadings and latent variables; all loadings are significant at ***p < .001. a = revised/modified items; re = reversed item; vas = visual analogue scale. Tests: AES = *Apathy Evaluation Scale*; CCSM = *DSM-5 Self-Rated Level 1 Cross-Cutting Symptom Measure*; Ex. Funct. = *Executive Functioning (adapted items);* IPSM = *Interpersonal Sensitivity Measure*; PANAS = *Positive and Negative Affect Schedule*; POMS = *Profile of Mood States*; Soc. Excl. = *Social Exclusion*; SUDS = *Subjective Units of Discomfort Scale*; WHO-DAS 2.0 = *World Health Organization Disability Assessment Schedule 2.0*

The final CFA model (Model 7) demonstrated excellent fit across the full sample, with the following robust indices: CFI = 0.990, TLI = 0.989, RMSEA = 0.037, and SRMR = 0.039. These results indicate that the model provides a strong representation of the latent structure and is suitable for cross-group comparisons. For detailed parameter estimates and other information, refer to Supplements S2c + d. For a visual representation of the model, see Fig. 2.

### General health cluster

To examine the structural validity of the General Health variables and its latent factors Physical Functioning (PhF), Mental Health (MeH), Functional Health limitations (FHL), Quality of Life (QoL) and Subjective Well-Being (SWB), a series of CFA and MGCFA models were estimated. Table 4 summarizes the fit indices of all tested models. The initial General Health baseline model (Model 1) showed already good fit. Still, one item was excluded from the model due to an R^2^ less than 0.20, leaving 19 items for further analysis. In addition, modification indices (MI) greater than 50 indicated the need for several adjustments to improve model fit. Details on all changes made can be found in General Health cluster supplement S3.

**Table 4:** General Health (MG)CFA Models in stepwise process

No	Model description	N (Sample Size)	CFI	TLI	RMSEA	SRMR	χ^2^(test [comp mod.]	ΔCFI
1	MGCFA Model	2616 (1597/1019)	0.961	0.954	0.074	0.081	-	-
2	CFA Model Healthy group	1597	0.984	0.980	0.040	0.052	-	-
3	CFA Model Clinical group	1019	0.992	0.990	0.044	0.048	-	-
4	MGCFA Configural Model	2616 (1597/1019)	0.992	0.991	0.042	0.048	-	-
5	MGCFA Metric Invariance Model	2616 (1597/1019)	0.977	0.973	0.062	0.066	χ^2^(296) = 317.41^**^[4]	0.016^a^
6	MGCFA Scalar Invariance Model	2616 (1597/1019)	0.976	0.971	0.062	0.067	χ^2^(310) = 130.79^**^[5]	0.001^b^
7	CFA Final General Health Model	2616	0.993	0.991	0.037	0.044	-	-
8	CFA One-Factor Model	2616	0.917	0.907	0.117	0.123	χ^2^(152) = 2341.20**[7]	-
9	CFA Orthogonal Factors Model	2616	0.346	0.254	0.331	0.359	χ^2^(150) = 2522.10**[7]	-

*CFA* Confirmatory Factor Analysis, *CFI* Comparative Fit Index, *Comp. mod.* compared model, *MGCFA* Multigroup Confirmatory Factor Analysis, *TFI* Tucker–Lewis Fit Index, *TLI* Tucker–Lewis Index, *RDoC* Research Domain Criteria, *RMSEA* Root Mean Square Error of Approximation, *SRMR* Standardized Root Mean Square Residual

All reported fit indices are based on robust estimators

^*^p < .05

^**^p < .01

^***^p < .001 (two-tailed)

ΔCFI values reflect comparisons to previous model steps as indicated in parentheses

^a^ΔCFI < .01 (acceptable threshold; Cheung & Rensvold, 2002)

^b^ΔCFI < .002 (strict criterion; Meade et al., 2008)

The revised configural model (Model 4) demonstrated excellent fit: CFI = 0.992, RMSEA = 0.042, and SRMR = 0.048. Metric invariance was not supported by the primary ΔCFI criterion, which exceeded the targeted threshold (ΔCFI = 0.016). Consequently, additional fit indices were examined to further contextualize the model fit and metric invariance. Notably, both RMSEA and SRMR improved from the configural to the metric model (ΔRMSEA = –0.024, ΔSRMR = –0.018), indicating that the constrained metric model provided a better absolute fit despite the slightly elevated ΔCFI. Scalar invariance was supported under both criteria. The final multidimensional model showed significantly better fit than both a unidimensional model and a model with orthogonal factors. A reduced “TOP-Item” model could not be estimated due to identification constraints.

**Fig. 3:**
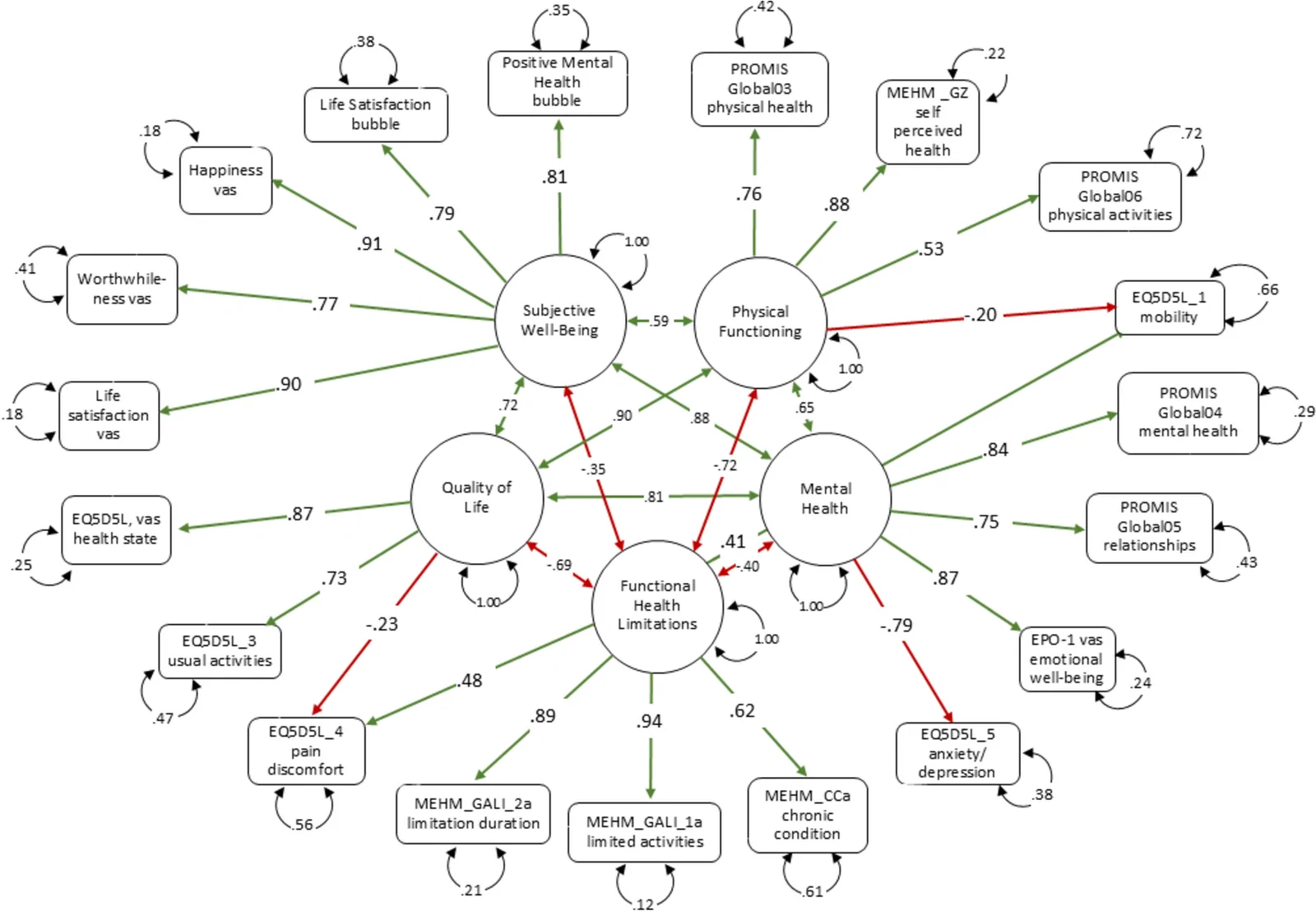
*Factorial loadings of the final model on the five General Health domains.* **Note.** Standardized factor loadings and latent variables; all loadings are significant at ***p < .001. a = revised/modified items; vas = visual analog scale; bubble = bubble item rating. Tests: EPO = Emotional and Psychological Outcome; EQ5D5L = EuroQol 5-Dimension 5-Level questionnaire; MEHM = Minimum European Health Module; PROMIS = PROMIS v1.2 Global-10, GMH2: Patient Reported Outcome Measurement Information System

The final CFA model (Model 7) for the General Health cluster demonstrated an excellent fit, with CFI = 0.993, RMSEA = 0.037, and SRMR = 0.044. Figure 3 visualizes the structural representation of the final model and detailed information including factor loadings and factor covariances can be found in supplements S3 c + d.

These comparisons confirm that the final model (Model 7) is the most accurate representation of the General Health constructs and outperforms simpler or theoretically less plausible alternatives.

### Clinical and psychosocial cluster

The evaluation of this variable cluster followed the established MGCFA framework. Fit indices and model comparisons are presented in Table 5. We assessed the configural invariance of the risk and protective factors model (Model 1) using all preselected items, including those moved from the RDoC cluster. The latent factors included Childhood Trauma (CTrauma), Trauma Experience (Trauma Exp), Trauma Sequelae (Trauma Seq), Depression (Depr), Anxiety (Anx), Stress, Eating Behavior (Eat), Somatization (SOM), Sense of Control (SoC), Social Well-Being/Functioning (Social), Emotion Regulation Suppression (Suppr), Emotion Regulation Reappraisal (Reapp), and Resilience (Resil).The initial model demonstrated good fit indices (N = 2588): CFI = 0.986, RMSEA = 0.032, and SRMR = 0.043. After the regular optimization procedure 60 items remained in the final model. However, exclusions were noted as a topic for further discussion in the Delphi process. A detailed depiction of all changes can be found in supplements S4.

**Table 5:** Clinical and Psychosocial Factors (MG)CFA Models in stepwise process

No	Model description	N (Sample Size)	CFI	TLI	RMSEA	SRMR	χ^2^(test [comp mod.]	ΔCFI
1	MGCFA Model0	2588 (1587/1001)	0.986	0.985	0.032	0.043	-	-
2	CFA Model Healthy group	1589	0.988	0.986	0.024	0.036	-	-
3	CFA Model Clinical group	1005	0.996	0.996	0.033	0.039	-	-
4	MGCFA Configural Model	2594 (1589/1005)	0.990	0.989	0.029	0.036	-	-
5	MGCFA Metric Invariance Model	2594 (1589/1005)	0.985	0.984	0.033	0.045	χ^2^(2772) = 214.84^**^(4)	0.006^a^
6	MGCFA Scalar Invariance Model	2594 (1589/1005)	0.985	0.984	0.033	0.045	χ^2^(2815) = 340.85^**^(5)	0.006^a^
7	CFA Final Clinical and Psychosocial Model	2594	0.992	0.992	0.026	0.033	-	-
8	CFA Final Model rep. sample	2283	0.990	0.989	0.026	0.034		
9	CFA One-Factor Model	2283	0.883	0.879	0.088	0.100	χ^2^(1890) = 7625.7^**^[8]	-
10	CFA Orthogonal Factors Model	2283	0.883	0.879	0.088	0.100	χ^2^(1890) = 7625.7^**^[8]	-

*CFA* Confirmatory Factor Analysis, *CFI* Comparative Fit Index, *Comp. mod.* compared model, *MGCFA* Multigroup Confirmatory Factor Analysis, *TFI* Tucker–Lewis Fit Index, *TLI* Tucker–Lewis Index, *RDoC* Research Domain Criteria, *RMSEA* Root Mean Square Error of Approximation, *SRMR* Standardized Root Mean Square Residual

All reported fit indices are based on robust estimators

^*^p < 0.05

^**^p < 0.01

^***^p < 0.001 (two-tailed)

ΔCFI values reflect comparisons to previous model steps as indicated in parentheses

^a^ΔCFI < 0.01 (acceptable threshold; Cheung & Rensvold, 2002)

^b^ΔCFI < 0.002 (strict criterion; Meade et al., 2008)

Configural invariance was established across both participant groups (healthy and those with a mental health diagnosis), with the configural model (Model 4) demonstrating excellent fit: CFI = 0.990, RMSEA = 0.029, SRMR = 0.036. Group-specific CFAs (Models 2 and 3) also demonstrated good to excellent model fit, further supporting configural invariance. Subsequent metric and scalar invariance testing showed a ΔCFI of 0.006 (Models 5/6), which fall below the commonly used threshold. Thus, metric and scalar invariance can be assumed under standard criteria.

Emphasis was placed on Model 8, which reflects the final Clinical And Psychosocial Factors model including the additional BRS items in the representative community sample (N = 2283). This model demonstrated excellent fit (CFI = 0.990, RMSEA = 0.026, SRMR = 0.034), with BRS items showing consistently high factor loadings ranging from 0.52 to 0.81. In comparison, CD-RISC items exhibited lower loadings (0.52 and 0.62). Due to the complexity of the final model, no path diagram is provided; however, full details on standardized factor loadings and inter-factor covariances are presented in Supplementary Tables S4c and S4d.

To further examine model parsimony, two theoretically relevant alternative models were tested in the representative community sample: a unidimensional (Model 9) and an orthogonal factors model (Model 10). Both models demonstrated substantially worse fit and chi-square difference tests confirmed significantly inferior model fit. These findings support the appropriateness of a multidimensional and correlated factor structure for modeling the Clinical and Psychosocial construct. A comparison to a TOP-item model was omitted due to several reasons, including the presence of single-item factor solutions, the lack of statistical comparability resulting from substantial differences in model complexity, and the unequal number of items across models.

## Discussion

The present study aimed to evaluate the latent structure and measurement invariance of three clusters in a MDS approach, namely RDoC, General Health, and Clinical and Psychosocial Factors. The study focused on its applicability across clinical and non-clinical populations. The findings suggest that while the RDoC and Clinical/Psychosocial models demonstrate acceptable configural, metric and scalar measurement invariance, the General Health model exhibited non-invariance at the metric level based on our primary ΔCFI criterion. These results have significant implications for using the MDS in comparative studies, highlighting where cross-group comparisons are fully justified and where caution is warranted due to potential measurement artifacts.

The initial models for all the three clusters showed acceptable fit, indicating generally plausible factor structures. Item-level diagnostics, including explained variance and modification indices, were subsequently used to identify opportunities for item reduction and model refinement. This analyses were embedded in the ongoing Delphi consensus process after each major analytical step, the multidisciplinary MDS Delphi group reviewed the findings and considered them alongside theoretical relevance and clinical utility. This combination of empirical evidence and expert judgement was essential for developing a parsimonious MDS without sacrificing conceptual coverage. Accordingly, some constructs such as substance use, were retained despite comparatively weaker statistical performance because of their clinical relevance.

Following item reduction, the final configural models showed very good fit in both the clinical and non-clinical groups. This finding indicates that the same broad factor structures were represented across the two populations. It is also consistent with previous research emphasizing the multidimensional character of mental health and related functional and psychosocial domains (e.g., [20]) Preserving this multidimensional structure appears particularly important for the Clinical and Psychosocial cluster, in which conceptually distinct but related constructs entail too much specific variance to be adequately represented by a single general factor. This interpretation was further supported by comparisons with alternative model specifications. Across all three clusters, the theoretically derived multidimensional models outperformed both unidimensional general-factor models and models in which the latent factors were constrained to be orthogonal. The final models therefore support not only the distinctiveness of the assessed constructs but also the meaningful relationships among them. These findings favor a multidimensional framework for representing mental health, general health, functioning, and psychological characteristics in heterogeneous populations.

Measurement invariance was evaluated primarily using the conventional criterion of ΔCFI < 0.01 [22]. The RDoC and Clinical/Psychosocial clusters met this criterion at the metric and scalar levels. These results indicate that factor loadings and item intercepts or thresholds were sufficiently comparable across clinical and non-clinical groups. Consequently, relationships between indicators and latent constructs can be interpreted similarly across groups, and comparisons of latent means are supported for these two clusters.

The General Health cluster did not meet the primary ΔCFI criterion at the metric level. Nevertheless, the pattern of findings was not uniformly indicative of substantial model deterioration: RMSEA and SRMR improved after equality constraints were introduced. Moreover, the large sample sizes may have increased sensitivity to relatively small discrepancies between groups, a known limitation of the CFI [23]. These observations suggest that the departure from metric invariance in the general health cluster may be limited rather than reflecting a fundamentally different construct structure across groups. However, because the prespecified criterion was exceeded, full metric invariance should not be claimed. Comparisons of associations or group means involving General Health should therefore be treated as provisional until the source and practical magnitude of the non-invariance have been examined more closely. Stricter criteria, such as ΔCFI < 0.002, have been proposed, but their application would have increased the likelihood of rejecting potentially useful models, particularly given the size and complexity of the present models. The conventional threshold was considered appropriate for this developmental study, whose purpose was to inform construction of a scalable MDS rather than to establish diagnostic equivalence. Even under this criterion, however, the General Health result demonstrates the importance of considering the complete pattern of fit indices rather than describing all three clusters as equally invariant.

Differences in the General Health factor loadings may arise because clinical and non-clinical participants interpret particular health-related indicators differently. Illness experience, current health status, health literacy, or group-specific response tendencies may influence the meaning or relevance of individual items. Future studies should identify the sources for the observed non-invariance and investigate whether revisions to item wording, response options, or contextual framing improve comparability without weakening construct validity.

Taken together, the findings provide preliminary evidence that the selected RDoC and Clinical/Psychosocial constructs can be assessed comparably in non-clinical participants and in individuals reporting a diagnosis or contact with mental health services. At the same time, the General Health findings illustrate the challenges of cross-group psychometric modeling, where strict measurement equivalence is often difficult to achieve [23].

Building upon the established configural invariance, the partial confirmation of metric invariance —specifically across the RDoC and the Clinical and Psychosocial clusters—indicates that the relationships between observed indicators and their latent constructs are comparable across clinical and non-clinical groups for these domains. This consistency allows for meaningful interpretation of factor loadings and supports the validity of cross-group comparisons using manifest questionnaire scores in these invariant clusters. Although scalar invariance does not guarantee the complete absence of bias, it suggests that observed group differences are not solely due to measurement artifacts. These results provide preliminary evidence that the selected constructs are applicable to both healthy controls (non-clinical) and self-reported diagnoses/help seeking (clinical) populations within the MDS framework and its intended use in heterogeneous settings within the DZPG.

### Methodological limitations

Despite these promising results, several methodological limitations should be acknowledged. First, the indicator variables deviated from a normal distribution. The WLSMV estimator was therefore used because of its robustness to non-normality. Although appropriate for the data, the choice of estimator must be considered when interpreting and comparing model-fit indices.

Second, item reduction and model refinements were partly data-driven. Explained variance (R^2^) and modification indices (MIs) were used to identify weak or overlapping indicators, with a MI threshold of 50 applied to focus on potentially consequential misspecifications. All proposed modifications were evaluated for theoretical plausibility, and the Delphi consensus group provided an additional conceptual safeguard. Nevertheless, model modifications based on the observed data may capitalize on sample-specific characteristics.

Relatedly, model development and validation were conducted within the same dataset. Although we utilized a confirmatory framework—including multi-group CFA across clinical and non-clinical populations—item selection and model refinement inevitably relied on data-driven decisions, which may inflate model fit limiting generalizability. Ideally, a split-sample approach would have been implemented, conducting model specification and refinement in an exploratory subsample before testing it in an independent holdout sample. The absence of such a cross-validation strategy is an important limitation that may have led to optimistic parameter estimates and model fit indices. Consequently, independent replication remains a critical next step. It is worth noting, however, that this constraint partly reflects the pragmatic nature of developing a brief MDS, where initial phases require iterative, data-driven item reduction to prioritize feasibility and measurement efficiency before large-scale cross-validation can be achieved.

Third, the classification of clinical and non-clinical participants was based on self-reported mental health histories and healthcare contact rather than clinician-administered diagnostic assessments. The clinical group may consequently have included clinical cases, help-seeking individuals and remitted patients. Conversely, the non-clinical group may have included participants with undetected or subthreshold psychopathology. Such misclassification could have reduced observable group differences and may have produced conservative estimates of non-invariance. Although this pragmatic classification is consistent with the aim of developing a scalable assessment, future validation trials should include rigorously characterized, clinician-confirmed samples.

Finally, while configural, metric, and scalar invariance were achieved based on moderate criteria, those definitions of invariance were not fully met in the General Health cluster. This underscores the necessity for cautious interpretation of cross-group comparisons and emphasizes the importance of further validation in diverse samples. The proposed measurement models' plausibility and generalizability must be confirmed by future external cross-validation, given the number of data-driven decisions and model modifications throughout the development process. Furthermore, future studies should not only re-evaluate construct validity but also address broader psychometric quality criteria—including predictive validity, test–retest reliability, and practical applicability in real-world clinical and research settings.

### Implications for minimum dataset development

The findings from these MGCFAs were systematically incorporated into the subsequent Delphi consensus process. For each cluster, a multidisciplinary expert panel was presented with detailed information on the final factor structures, standardized factor loadings of the retained items, and analyses of “top-item” models containing the highest-loading indicator or indicators for each factor. Full overviews of the selected Top Variables per cluster are available in Table 1. These results informed targeted recommendations for item selection and were discussed during the Delphi consensus meetings, alongside clinical relevance, conceptual coverage, participant burden, and practical feasibility. Based on these results, a preliminary MDS proposal comprising approximately 130 items, including the sociodemographic section, was submitted to the group for consent. Although the proposed item sets were broadly supported, domains such as substance use and trauma required further conceptual discussion. The meeting therefore represented one stage of an iterative consensus process rather than its conclusion.

Future versions of the MDS should continue to balance structural validity and reliability against brevity and ease of implementation. Ongoing development should incorporate external validation, stakeholder and patient and public involvement, and adaptation to changing scientific and clinical requirements. The objective is not merely to minimize the number of items, but to establish an assessment framework that permits efficient and meaningful data collection across diverse research and healthcare contexts.

This study provides initial evidence that the RDoC and Clinical/Psychosocial clusters of the proposed MDS have comparable factor structures, loadings, and item thresholds across clinical and non-clinical populations. The General Health cluster showed a stable configural structure but did not meet the primary criterion for metric invariance. Cross-group comparisons involving this cluster therefore require caution and further investigation.

The analyses provided an empirical foundation for the subsequent Delphi consensus process by identifying indicators that combined statistical performance with conceptual and clinical relevance. By demonstrating both psychometric rigor within a developmental MDS framework and practical feasibility, this study contributes to the ongoing standardization of mental health assessments and highlights the MDS’s relevance as a scalable framework for heterogeneous research and care settings. The development of a harmonized MDS is particularly crucial in the context of the DZPG, which aims to establish integrative, evidence-based standards across multiple institutions and populations. The DZPG MDS offers a shared measurement backbone that can enhance comparability, data integration, and collaborative research efforts within and beyond the DZPG. Independent replication, external cross-validation, and continued refinement will be necessary to ensure that the DZPG MDS remains psychometrically sound, clinically meaningful, and feasible to implement.

## Supplementary Information

Below is the link to the electronic supplementary material.

Supplementary file1 (DOCX 41 KB)

Supplementary file2 (DOCX 34 KB)

Supplementary file3 (DOCX 30 KB)

Supplementary file4 (DOCX 46 KB)

## Data Availability

All original data are on record and accessible to inspection by request.

## Code availability

Available from the authors upon request.
