# Supplementary material for: Psychometric validation of research domain criteria, general health, and clinical-psychosocial parts of the minimum dataset of the German Center for Mental Health—informing a Delphi process
Source: Eur Arch Psychiatry Clin Neurosci. 2026 Aug 22;276(7):3563–82. doi: 10.1007/s00406-026-02315-w (PMC13562200; doi:10.1007/s00406-026-02315-w)
Supplement: Supplementary file 1 [file 406_2026_2315_MOESM1_ESM.docx]

**Supplement S1: Detailed steps of the MGFCA procedure**

The following MGCFA steps were applied consistently across the three clusters—RDoC, General Health, and Clinical and Psychosocial Factors:

1. **Configural invariance**
   - A baseline model with all preselected variables was specified for both groups (non-Clinical vs. Clinical/Helpseeking).
   - Items/scales with R² < .20 in both groups were removed (optimization of explanatory power and model fit); if values differed, the item was retained.
   - Modification indices > 50 were reviewed, with theoretical considerations guiding item removal or reassignment.
   - No covariances between items/scales were added.
   - The final configural model was tested separately in both groups, then across groups.
2. **Metric invariance**
   - Factor loadings were constrained to equality across groups.
   - Model fit was compared with the configural model using the appropriate chi-square difference tests and ΔCFI.
   - Thresholds: ΔCFI < .01 (standard) [1], ΔCFI < .002 (stricter) [2, 3].
3. **Scalar invariance**
   - Factor loadings and intercepts were constrained to equality.
   - Model fit was compared with the metric model (appropriate chi-square, ΔCFI).
   - Same thresholds were applied.
   - After this step, a final model per cluster (without group comparison) was specified.
4. **Reduced-item models**
   - Final models were compared with reduced item versions (TOP3 for RDoC; TOP1 for Global Health; combinations for Clinical und Psychosocial Factors).
   - Item selection was based on factor loadings and expert content validity ratings from the Delphi process.
   - Two alternative models were also tested: (1) a single-factor model, and (2) independent factors without covariances to unsure that the final model factor structure provided a superior fit compared to more constrained or less structured alternatives.

**Deviations from procedure**

- Some variables (e.g., CTS [4], ERQ [5], IPSM [6]) were reassigned from RDoC to the Clinical and Psychosocial cluster after expert review.
- For resilience (within the Clinical and Psychosocial cluster), the BRS assessment was not available in the clinical sample. Therefore, a separate analysis was conducted in the representative sample only to compare BRS and CD-RISC items and identify the most suitable measures.

**References**

1.	Cheung GW, Rensvold RB (2002) Evaluating Goodness-of-Fit Indexes for Testing Measurement Invariance. Structural Equation Modeling 9(2):233–255. https://doi.org/10.1207/S15328007SEM0902_5

2.	Meade AW, Johnson EC, Braddy PW (2008) Power and sensitivity of alternative fit indices in tests of measurement invariance. The Journal of applied psychology 93(3):568–592. https://doi.org/10.1037/0021-9010.93.3.568

3.	Putnick DL, Bornstein MH (2016) Measurement Invariance Conventions and Reporting: The State of the Art and Future Directions for Psychological Research. Developmental review : DR 41:71–90. https://doi.org/10.1016/j.dr.2016.06.004

4.	Grabe HJ, Schulz A, Schmidt CO, Appel K, Driessen M, Wingenfeld K, Barnow S, Spitzer C, John U, Berger K, Wersching H, Freyberger HJ (2012) Ein Screeninginstrument für Missbrauch und Vernachlässigung in der Kindheit: der Childhood Trauma Screener (CTS) (A brief instrument for the assessment of childhood abuse and neglect: the childhood trauma screener (CTS)). Psychiatrische Praxis 39(3):109–115. https://doi.org/10.1055/s-0031-1298984

5.	Abler B, Kessler H (2009) Emotion Regulation Questionnaire – Eine deutschsprachige Fassung des ERQ von Gross und John. Diagnostica 55(3):144–152. https://doi.org/10.1026/0012-1924.55.3.144

6.	Boyce P, Parker G (1989) Development of a scale to measure interpersonal sensitivity. The Australian and New Zealand journal of psychiatry 23(3):341–351. https://doi.org/10.3109/00048678909068291

Förstner, B.R., Böttger, S.J., Seegert, T., Kerber, A., Terhorst, Y., Heinz, A., Dziobek, I., Voigt, B., Schneider, S., Opel, N., Walter, M., Braun, U., Meyer-Lindenberg, A., Koutsouleris, N., Falkai, P.Schmitz, N., Fallgatter, A.J., Sünkel, U., Lipinski, S., Rapp, M.A., Tschorn, M. + MDS Expert Group -

Psychometric validation of Research Domain Criteria, General Health, and Clinical-Psychosocial parts of the Minimum Dataset of the German Center for Mental Health - Informing a Delphi Process

European Archives of Psychiatry and Clinical Neuroscience

Corresponding Author: Bernd R Förstner bernd.foerstner@uni-potsdam.de
