# Supplementary material for: Psychometric validation of research domain criteria, general health, and clinical-psychosocial parts of the minimum dataset of the German Center for Mental Health—informing a Delphi process
Source: Eur Arch Psychiatry Clin Neurosci. 2026 Aug 22;276(7):3563–82. doi: 10.1007/s00406-026-02315-w (PMC13562200; doi:10.1007/s00406-026-02315-w)
Supplement: Supplementary file 2 [file 406_2026_2315_MOESM2_ESM.docx]

**Supplements S2: RDoC cluster**

**Table S2a: Removed Items from RDoC Model1**

| Variable | Domain | R² - Healthy group | R²- Helpseeking / Diagnosis group |
| --- | --- | --- | --- |
| PID5 Item 64 | NVS | .147 | .100 |
| CTS Item1 | NVS | .099 | .114 |
| CTS Item2 | NVS | .040 | .029 |
| CTS Item 3 | NVS | .103 | .145 |
| CTS Item 4 | NVS | .041 | .037 |
| CTS Item 5 | NVS | .002 | .046 |
| Social Discrimination | SoP | .167 | .164 |
| Social support | SoP | .120 | .151 |
| Social support (Bubble) | SoP | .130 | .204* |
| IPSM Item 30 | SoP | .161 | .265* |
| IPSM Item 33 | SoP | .101 | .168 |
| IPSM Item 36 | SoP | .106 | .155 |
| LPFS-BF2.0 Item 7 | SoP | .215* | .164 |
| ERQ Item 7 | SoP | .000 | .068 |
| ERQ Item 8 | SoP | .001 | .043 |
| ERQ Item 2 | SoP | .112 | .076 |
| ERQ Item 6 | SoP | .049 | .049 |
| LHA Item 4 | SoP | .019 | .009 |
| AES Item 10 | SMS | .183 | .281* |

**Note.** Tests: AES: Apathy Evaluation Scale; CTS: Childhood Trauma Screener; ERQ: Emotion Regulation Questionnaire; IPSM: Interpersonal Sensitivity Measure; LHA: Life History of Aggression; LPFS-BF 2.0: Level of Personality Functioning Scale - Brief Form 2.0; PID-5: Personality Inventory for DSM-5; Domain: NVS: Negative Valence Systems, SoP: Social Processes, SMS: Sensorimotor Systems.

*R² values > .20; in further steps, these items were initially retained due to group congruence but excluded in subsequent steps due to consistently low R² or modification index issues.

15 Items: CTS, ERQ, Social support, IPSM were moved to the clinical and psychosocial factors cluster
+ LHA_4a got removed because of high missing rate (WLSMV)

**Table S2b: Modification indices considered in model specification**

| No. | **lhs** | **op** | **rhs** | **MI** | **EPC** | **decision** |
| --- | --- | --- | --- | --- | --- | --- |
| 1 | PVS | =~ | POMS vigorous | 236.810 | -0.621 | moved to PVS |
| 2 | SoP | =~ | CCSM_1 anhedonia | 529.490 | 1.046 | unspecific variable  -> deleted |
|  | NVS | =~ | CCSM_1 anhedonia | 513.933 | -0.792 |  |
|  | CoS | =~ | CCSM_1 anhedonia | 208.597 | 0.542 |  |
|  | ARS | =~ | CCSM_1 anhedonia | 163.995 | 0.618 |  |
| 3 | PANAS irritable | ~~ | CCSM_3 | 96.136 | -0.228 | Irritability congruence not considered due to allocation to different factors to maintain theoretical distinction. |

**Note.** lhs = dependent variable, op = operator (~~ covariance, ~ regression), rhs = independent variable, MI = Modification Index, EPC = Expected Parameter Change. Tests: CCSM: DSM-5 Self-Rated Level 1 Cross-Cutting Symptom Measure; POMS: Profile of Mood States; Domain: NVS: Negative Valence Systems, SoP: Social Processes.
All other modification indices were either theoretically unjustified, did not meaningfully improve model fit, or would have resulted in model overfitting.

**TableS2c: Final RDoC (Model7) Item Factor loadings**

| **Latent Factor** | **Indicator** | **B** | **SE** | **Z** | **Beta** | **sig** |
| --- | --- | --- | --- | --- | --- | --- |
| PVS | PANAS_active | 0.693 | 0.019 | 36.814 | .693 | *** |
| PVS | PANAS_interested | 0.786 | 0.018 | 43.435 | .787 | *** |
| PVS | PANAS_excited | 0.702 | 0.017 | 40.142 | .702 | *** |
| PVS | PANAS_determined | 0.760 | 0.017 | 43.988 | .760 | *** |
| PVS | POMS_vigorous | 0.745 | 0.018 | 40.836 | .746 | *** |
| NVS | vas_SUDS | 0.634 | 0.018 | 35.785 | .634 | *** |
| NVS | CCSM_6 anxiety | 0.828 | 0.015 | 53.867 | .827 | *** |
| NVS | CCSM_7 panic | 0.760 | 0.021 | 35.650 | .760 | *** |
| NVS | CCSM_8a agoraphobia | 0.696 | 0.021 | 33.032 | .696 | *** |
| NVS | PANAS_ashamed | 0.639 | 0.024 | 26.731 | .639 | *** |
| NVS | PANAS_irritable | 0.637 | 0.019 | 32.721 | .637 | *** |
| CoS | CCSM_15_re memory | 0.778 | 0.023 | 33.871 | .778 | *** |
| CoS | Ex. Funct._a1_m memory | -0.703 | 0.019 | -37.931 | -.703 | *** |
| CoS | Ex. Funct._a2_m flexibitlity | -0.700 | 0.020 | -35.010 | -.700 | *** |
| CoS | Ex. Funct._a3_m planning | -0.766 | 0.020 | -37.868 | -.766 | *** |
| SoP | WHODAS20_11_r friendships | 0.579 | 0.019 | 30.057 | .579 | *** |
| SoP | CCSM_1a activities with others | -0.795 | 0.016 | -50.079 | -.794 | *** |
| SoP | Vas Social Exclusion | -0.677 | 0.019 | -35.773 | -.677 | *** |
| SoP | IPSM_24 self doubt | -0.610 | 0.020 | -31.106 | -.610 | *** |
| SoP | CCSM_3 irritability | -0.737 | 0.017 | -42.661 | -.737 | *** |
| SMS | AES_3 autonomy | 0.584 | 0.023 | 24.910 | .584 | *** |
| SMS | AES_17 initiative | 0.848 | 0.019 | 43.934 | .848 | *** |
| SMS | AES_2 productivity | 0.708 | 0.021 | 33.294 | .709 | *** |
| SMS | AES_16 goal orientation | 0.531 | 0.024 | 22.442 | .531 | *** |
| ARS | CCSM_14_r sleep | 0.667 | 0.019 | 35.418 | .667 | *** |
| ARS | POMS_exhausted | -0.871 | 0.013 | -69.381 | -.870 | *** |
| ARS | POMS fatigued | -0.790 | 0.015 | -52.272 | -.790 | *** |
| ARS | POMS_worn out | -0.854 | 0.015 | -58.839 | -.854 | *** |

**Note**. RDoC Domain: ARS: Arousal and Regulatory Systems; CoS: Cognitive Systems; NVS: Negative Valence Systems; PVS: Positives Valence Systems; SMS: Sensorimotor Systems; SoP: Social Processes. Test: AES: Apathy Evaluation Scale; Ex.Funct.: Executive Functioning (adapted/modified items BRIEF); CCSM: DSM-5 Self-Rated Level 1 Cross-Cutting Symptom Measure; PANAS: Positive and Negative Affect Schedule; POMS: Profile of Mood States; SUD: Subjective Units of Discomfort; WHO-DAS 2.0: World Health Organization Disability Assessment Schedule 2.0.

a = modified item; r = reversed item; m = item reworded with reference to a different questionnaire

**Table S2d: Final RDoC CFA (Model7) factor covariance matrix**

|  | **PVS** | **NVS** | **CoS** | **SoP** | **SMS** | **ARS** |
| --- | --- | --- | --- | --- | --- | --- |
| **PVS** | 1.000 |  |  |  |  |  |
| **NVS** | -0.595 | 1.000 |  |  |  |  |
| **CoS** | 0.577 | -0.741 | 1.000 |  |  |  |
| **SoP** | 0.706 | -0.940 | 0.742 | 1.000 |  |  |
| **SMS** | 0.689 | -0.498 | 0.520 | 0.600 | 1.000 |  |
| **ARS** | 0.682 | -0.771 | 0.646 | 0.789 | 0.514 | 1.000 |

**Note.** Latent Factors: ARS: Arousal and Regulatory Systems; CoS: Cognitive Systems; NVS: Negative Valence Systems; PVS: Positives Valence Systems; SMS: Sensorimotor Systems; SoP: Social Processes.

Förstner, B.R., Böttger, S.J., Seegert, T., Kerber, A., Terhorst, Y., Heinz, A., Dziobek, I., Voigt, B., Schneider, S., Opel, N., Walter, M., Braun, U., Meyer-Lindenberg, A., Koutsouleris, N., Falkai, P.Schmitz, N., Fallgatter, A.J., Sünkel, U., Lipinski, S., Rapp, M.A., Tschorn, M. + MDS Expert Group -

Psychometric validation of Research Domain Criteria, General Health, and Clinical-Psychosocial parts of the Minimum Dataset of the German Center for Mental Health - Informing a Delphi Process

European Archives of Psychiatry and Clinical Neuroscience

Corresponding Author: Bernd R Förstner bernd.foerstner@uni-potsdam.de
