# Supplementary material for: Psychometric validation of research domain criteria, general health, and clinical-psychosocial parts of the minimum dataset of the German Center for Mental Health—informing a Delphi process
Source: Eur Arch Psychiatry Clin Neurosci. 2026 Aug 22;276(7):3563–82. doi: 10.1007/s00406-026-02315-w (PMC13562200; doi:10.1007/s00406-026-02315-w)
Supplement: Supplementary file 3 [file 406_2026_2315_MOESM3_ESM.docx]

**Supplements S3: General Health cluster**

**Table S3a: Removed Items from RDoC Model1**

| Variable | Domain | R² - Healthy group | R²- Helpseeking / Diagnosis group |
| --- | --- | --- | --- |
| EQ5D5L Item 2 | QoL | .069 | .117 |

**Note.** Tests: EQ5D5L: EuroQol 5-Dimension 5-Level questionnaire; Domain: QoL: Quality of Life.

**Table S3b: Modification indices considered in model specification**

| No. | **lhs** | **op** | **rhs** | **MI** | **EPC** | **decision** |
| --- | --- | --- | --- | --- | --- | --- |
| 1 | PhF | =~ | PROMIS Global 6 | 178.044 | -0.499 | moved to GPH from FHL |
| 2 | MeH | =~ | EQ5D5L, Item 4 | 325.523 | -0.647 | moved to GMH from QoL |
| 3 | FHL | =~ | EQ5D5L, Item 1 | 265.400 | 0.602 | moved to FHL and GPH from QoL |
|  | PhF | =~ | EQ5D5L, Item 1 | 78.184 | 0.679 |  |
| 4 | FHL | =~ | EQ5D5L, Item 5 | 504.111 | -0.827 | additional factor loading on FHL |

**Note.** lhs = dependent variable, op = operator (~~ covariance, ~ regression), rhs = independent variable, MI = Modification Index, EPC = Expected Parameter Change. FHL: Functional Health Limitations; MeH: Mental Health; PhF: Physical Functioning.
All other modification indices were either theoretically unjustified, did not meaningfully improve model fit, or would have resulted in model overfitting.

**TableS3c: Final General Health (Model7) Item Factor loadings**

| **Latent Factor** | **Indicator** | **B** | **SE** | **Z** | **Beta** | **sig** |
| --- | --- | --- | --- | --- | --- | --- |
| PhF | PROMIS Global03 physical health | .763 | 0.019 | 40.760 | .763 | *** |
| PhF | MEHM_GZ self percieved health | .739 | 0.017 | 44.247 | .882 | *** |
| PhF | PROMIS Global06 physical activities | .533 | 0.027 | 19.440 | .533 | *** |
| PhF | EQ5D5L Item1 mobility | -.203 | 0.043 | -4.726 | -.203 | *** |
| MeH | PROMIS Global04 mental health | .844 | 0.014 | 58.378 | .844 | *** |
| MeH | PROMIS Global05 relationships | .753 | 0.016 | 46.146 | .753 | *** |
| MeH | EPO-1 emotional well being | .869 | 0.017 | 50.175 | .869 | *** |
| MeH | EQ5D5L Item5 Anxiety/depression | -.789 | 0.020 | -38.791 | -.789 | *** |
| FHL | MEHM CCr chronic condition | .623 | 0.016 | 38.677 | .623 | *** |
| FHL | MEHM GALI 1a limited activitiest | .940 | 0.019 | 49.249 | .940 | *** |
| FHL | MEHM GALI 2a limitation duration | .889 | 0.012 | 73.079 | .889 | *** |
| FHL | EQ5D5L Item1 mobility | .421 | 0.041 | 10.201 | .420 | *** |
| FHL | EQ5D5L Item4 pain discomfort | .484 | 0.030 | 16.300 | .484 | *** |
| QoL | EQ5D5L Item3r usual activities | .727 | 0.027 | 27.022 | .726 | *** |
| QoL | EQ5D5L Item4 pain discomfort | -.226 | 0.032 | -7.034 | -.225 | *** |
| QoL | EQ5D5L health status vas | .865 | 0.020 | 42.246 | .865 | *** |
| SWB | Life Satisfaction vas | .904 | 0.017 | 54.649 | .904 | *** |
| SWB | Worthiness vas | .767 | 0.019 | 40.274 | .767 | *** |
| SWB | Happiness vas | .907 | 0.016 | 57.447 | .907 | *** |
| SWB | Life Satisfaction bubble | .786 | 0.015 | 50.856 | .787 | *** |
| SWB | Positve Mental Health bubble | .806 | 0.015 | 52.368 | .806 | *** |

**Note.** Latent Factors: FHL: Functional health Limitations; MeH: Mental Health; PhF: Physical Functioning; SWB: Subjective Well-Being; QoL: Quality of Life. Test: EPO = Emotional and Psychological Outcome; EQ5D5L = EuroQol 5-Dimension 5-Level questionnaire; MEHM = Minimum European Health Module; PROMIS = PROMIS v1.2 Global-10, GMH2: Patient Reported Outcome Measurement Information System. * p < .05, ** p < .01, *** p < .001 (two-tailed). a = modified item; r = reversed item; m = item reworded with reference to a different questionnaire; vas= Visual Analog Scale (new item); bubble = Bubble Rating (new item)

**Table S3d: Final General Health CFA (Model7) factor covariance matrix**

|  | **PhF** | **MeH** | **FHL** | **QoL** | **SWB** |
| --- | --- | --- | --- | --- | --- |
| **PhF** | 1.000 |  |  |  |  |
| **MeH** | .651 | 1.000 |  |  |  |
| **FHL** | -.719 | -.401 | 1.000 |  |  |
| **QoL** | .899 | .812 | -.692 | 1.000 |  |
| **SWB** | .586 | .875 | -.346 | .713 | 1.000 |

**Note.** Latent Factors: FHL: Functional health Limitations; MeH: Mental Health; PhF: Physical Functioning; SWB: Subjective Well-Being; QoL: Quality of Life.

Förstner, B.R., Böttger, S.J., Seegert, T., Kerber, A., Terhorst, Y., Heinz, A., Dziobek, I., Voigt, B., Schneider, S., Opel, N., Walter, M., Braun, U., Meyer-Lindenberg, A., Koutsouleris, N., Falkai, P.Schmitz, N., Fallgatter, A.J., Sünkel, U., Lipinski, S., Rapp, M.A., Tschorn, M. + MDS Expert Group -

Psychometric validation of Research Domain Criteria, General Health, and Clinical-Psychosocial parts of the Minimum Dataset of the German Center for Mental Health - Informing a Delphi Process

European Archives of Psychiatry and Clinical Neuroscience

Corresponding Author: Bernd R Förstner bernd.foerstner@uni-potsdam.de
