# Supplementary material for: Psychometric validation of research domain criteria, general health, and clinical-psychosocial parts of the minimum dataset of the German Center for Mental Health—informing a Delphi process
Source: Eur Arch Psychiatry Clin Neurosci. 2026 Aug 22;276(7):3563–82. doi: 10.1007/s00406-026-02315-w (PMC13562200; doi:10.1007/s00406-026-02315-w)
Supplement: Supplementary file 4 [file 406_2026_2315_MOESM4_ESM.docx]

**Supplements S4: Clinical and Psychosocial Factors cluster**

**Table S4a: Removed Items from Clinical and Psychosocial Factors Model1**

| Variable | Domain | R² - Healthy group | R²- Helpseeking / Diagnosis group |
| --- | --- | --- | --- |
| CCSM_21 | SubUse | .193 | .061 |
| CCSM_22 | SubUse | .040 | .188 |
| CCSM_23a | SubUse | .358* | .288* |
| CCSM_23a2 | SubUse | .301* | .423* |
| LEC5_a2 | TraumaExp | .294* | .150 |
| CTS_5 | CTrauma | .018 | .165 |
| EAT26D_23 | Eat | .230* | .137 |
| SNAQ_3 | Eat | .170 | .150 |
| Social support | Social | .065 | .133 |
| Social support bubble | Social | .080 | .198 |
| IPSM_30 | Social | .194 | .336* |
| IPSM_33 | Social | .159 | .250* |
| IPSM_36 | Social | .162 | .213* |

**Note.** Tests: CCSM: DSM-5 Level 1 Cross-Cutting Symptom Measure; CTS: Childhood Trauma Screener; EAT: Eating behavior, *EAT-26* Eating Attitudes Test; IPSM: Interpersonal Sensitivity Measure; LEC-5: Life Events Checklist for DSM-5; SNAQ: Short Nutritional Assessment Questionnaire. Domain: CTrauma: Childhood Trauma; Eat: Eating Behaviour; Social: Social Well-Being (functioning) SubUse: Substance Use; TraumaExp: Trauma Experience.

*R² values > .20; in further steps, these items were initially retained due to group congruence but excluded in subsequent steps due to consistently low R² or modification index issues or for SubUse moved to the sociodemographics section.

**Table S2b: Modification indices considered in model specification**

| No. | **lhs** | **op** | **rhs** | **MI** | **EPC** | **decision** |
| --- | --- | --- | --- | --- | --- | --- |
| 1 | IPSM_30 | ~~ | IPSM_36 | 502.606 | -.461 | IPSM_36 removed |
| 2 | TraumaSeq  CTrauma  Social  Anx  Stress  Depr | =~ | LEC_3a | 262.458  247.634  244.055  242.215  240.321  239.249 | .275  -.289  .162  .138  .136  .132 | removed unspecific item |
| 3 | TraumaExp | =~ | CTS_4 | 171.249 | -.290 | moved, removed by later decision |
| 4 | Depr | =~ | Loneliness | 174.982 | -.515 | additional factor laoding |

**Note.** lhs = dependent variable, op = operator (~~ covariance, ~ regression), rhs = independent variable, MI = Modification Index, EPC = Expected Parameter Change. Tests: CTS: Childhood Trauma Screener; IPSM: Interpersonal Sensitivity Measure; LEC-5: Life Events Checklist for DSM-5; Loneliness: Loneliness, SI Direct. Domain: *Anx* Anxiety, CTrauma: Childhood Trauma; Depr*:* Depression; Social: Social Well-Being (functioning); Trauma Exp: Trauma Experience; TraumaSeq: Trauma Sequelae.
All other modification indices were either theoretically unjustified, did not meaningfully improve model fit, or would have resulted in model overfitting.

**TableS4c: Final Clinical and Psychosocial Factors (Model7) Item Factor loadings**

| **Latent Factor** | **Indicator** | **B** | **SE** | **Z** | **Beta** | **sig** |
| --- | --- | --- | --- | --- | --- | --- |
| CTrauma | CTS_1 | 0.649 | 0.030 | 21.464 | .662 | *** |
| CTrauma | CTS_2 | -0.590 | 0.039 | -15.296 | -.591 | *** |
| CTrauma | CTS_3 | -0.800 | 0.033 | -24.096 | -.842 | *** |
| TraumaExp | LEC5_a1 | 0.772 | 0.041 | 19.038 | .787 | *** |
| TraumaExp | LEC5_a2 | 0.429 | 0.031 | 13.763 | .442 | *** |
| TraumaSeq | PCPTSD5_0 | 1.019 | 0.012 | 88.593 | 1.038 | *** |
| TraumaSeq | PCPTSD5_1 | -0.963 | 0.015 | -62.494 | -.964 | *** |
| TraumaSeq | PCPTSD5_2 | -0.963 | 0.015 | -62.397 | -.967 | *** |
| TraumaSeq | PCPTSD5_3 | -0.968 | 0.015 | -65.133 | -.968 | *** |
| TraumaSeq | PCPTSD5_4 | -0.927 | 0.016 | -58.817 | -.920 | *** |
| TraumaSeq | PCPTSD5_5 | -0.973 | 0.015 | -66.421 | -.971 | *** |
| Stress | DASS21_1 | 0.674 | 0.023 | 29.138 | .754 | *** |
| Stress | DASS21_6 | 0.670 | 0.022 | 31.024 | .722 | *** |
| Stress | DASS21_8 | 0.711 | 0.019 | 36.448 | .781 | *** |
| Stress | DASS21_11 | 0.649 | 0.022 | 30.087 | .682 | *** |
| Stress | DASS21_12 | 0.735 | 0.018 | 40.361 | .800 | *** |
| Stress | DASS21_14 | 0.659 | 0.025 | 26.534 | .705 | *** |
| Stress | DASS21_18 | 0.697 | 0.021 | 32.674 | .755 | *** |
| Stress | b_DASS21 | 0.540 | 0.014 | 38.097 | .741 | *** |
| Anx | DASS21_2 | 0.517 | 0.027 | 19.256 | .538 | *** |
| Anx | DASS21_4 | 0.539 | 0.030 | 17.860 | .580 | *** |
| Anx | DASS21_7 | 0.495 | 0.030 | 16.245 | .555 | *** |
| Anx | DASS21_9 | 0.647 | 0.026 | 25.111 | .723 | *** |
| Anx | DASS21_15 | 0.598 | 0.029 | 20.316 | .695 | *** |
| Anx | DASS21_19 | 0.605 | 0.026 | 23.534 | .646 | *** |
| Anx | DASS21_20 | 0.644 | 0.027 | 23.895 | .730 | *** |
| Anx | DASS21 anx bubble | 0.603 | 0.024 | 24.695 | .649 | *** |
| Anx | PHQ4_3 | 0.677 | 0.022 | 31.152 | .763 | *** |
| Anx | PHQ4_4 | 0.720 | 0.022 | 32.836 | .812 | *** |
| Anx | DSM5CC_6 | 0.725 | 0.017 | 42.622 | .780 | *** |
| Depr | DASS21_3 | 0.687 | 0.023 | 29.617 | .782 | *** |
| Depr | DASS21_5 | 0.695 | 0.020 | 35.612 | .740 | *** |
| Depr | DASS21_10 | 0.711 | 0.022 | 32.620 | .803 | *** |
| Depr | DASS21_13 | 0.784 | 0.018 | 42.972 | .851 | *** |
| Depr | DASS21_16 | 0.724 | 0.022 | 33.454 | .798 | *** |
| Depr | DASS21_17 | 0.709 | 0.022 | 31.894 | .795 | *** |
| Depr | DASS21_21 | 0.618 | 0.027 | 23.093 | .718 | *** |
| Depr | DASS21 Depr bubble | 0.746 | 0.018 | 40.372 | .797 | *** |
| Depr | PHQ4_1 | 0.704 | 0.020 | 34.398 | .765 | *** |
| Depr | PHQ4_2 | 0.728 | 0.021 | 35.001 | .819 | *** |
| Depr | CCSM_1 | 0.705 | 0.018 | 39.807 | .772 | *** |
| Resil | CDRISC2_1 | 0.516 | 0.025 | 20.728 | .536 | *** |
| Resil | CDRISC2_8 | 0.618 | 0.022 | 28.024 | .650 | *** |
| Resil | BRS_1 | 0.706 | 0.024 | 29.682 | .707 | *** |
| Resil | BRS_2 | -0.684 | 0.026 | -26.686 | -.683 | *** |
| Resil | BRS_3 | 0.520 | 0.026 | 20.012 | .520 | *** |
| Resil | BRS_4 | -0.668 | 0.022 | -30.040 | -.668 | *** |
| Resil | BRS_5 | 0.701 | 0.022 | 31.435 | .701 | *** |
| Resil | BRS_6 | -0.806 | 0.020 | -41.306 | -.805 | *** |
| Som | CCSM_9 | 0.655 | 0.022 | 29.717 | .682 | *** |
| Som | CCSM_10 | 0.779 | 0.025 | 31.694 | .826 | *** |
| Eat | EAT26D_23 | 0.984 | 0.013 | 74.971 | 1.000 | *** |
| SoC | SoC_1 | 0.821 | 0.020 | 41.839 | .853 | *** |
| SoC | SoC_2 | 0.805 | 0.020 | 39.616 | .835 | *** |
| ReApp | ERQ_7 | 0.696 | 0.036 | 19.126 | .710 | *** |
| ReApp | ERQ_8 | 0.814 | 0.038 | 21.231 | .833 | *** |
| Suppr | ERQ_2 | 0.931 | 0.044 | 21.323 | .950 | *** |
| Suppr | ERQ_6 | 0.713 | 0.036 | 19.619 | .728 | *** |
| Social | Loneliness SIDF | 0.389 | 0.040 | 9.823 | .406 | *** |
| Social | SocDis vas | 10.484 | 0.612 | 17.131 | .544 | *** |
| Social | IPSM_24 | 0.640 | 0.022 | 28.740 | .680 | *** |
| Social | IPSM_30 | 0.490 | 0.021 | 23.841 | .504 | *** |
| Social | IPSM_33 | 0.437 | 0.022 | 19.887 | .448 | *** |
| Depr | Loneliness SIDF | 0.265 | 0.039 | 6.864 | .277 | *** |

**Note.** Latent Factors: Ctrauma: Childhood Trauma; TraumaExp: Trauma Exposure; TraumaSeq: Trauma Sequelae; Stress: Stress; ANX: Anxiety; Depr: Depression; Resil: Resilience; Som: Somatization; Eat: Eating Behavior; SoC: Sense of Control; ReApp: Reappraissal; Suppr: Suppression; Social: Social Support. Tests: BRS = Brief Resilience Scale; CCSM = DSM-5 Self-Rated Level 1 Cross-Cutting Symptom Measure; CD-RISC-2 = Connor–Davidson Resilience Scale, 2-Item Version; DASS-21 = Depression Anxiety Stress Scales – 21 Item Version; EAT-26D = Eating Attitudes Test-26 (German version); ERQ = Emotion Regulation Questionnaire; IPSM = Interpersonal Sensitivity Measure; PHQ-4 = Patient Health Questionnaire-4; SoC = Sense of Control; SocDis = Social Discrimination Visual Analog Scale.

* p < .05, ** p < .01, *** p < .001 (two-tailed); Beta = standardized beta, vas= Visual Analog Scale (new item); bubble = Bubble Rating (new item)

**Table S4d: Final General Health CFA (Model7) factor covariance matrix**

| Factors | 1 | 2 | 3 | 4 | 5 | 6 | 7 | 8 | 9 | 10 | 11 | 12 | 13 |
| --- | --- | --- | --- | --- | --- | --- | --- | --- | --- | --- | --- | --- | --- |
| 1. CTrauma | 1.000 |  |  |  |  |  |  |  |  |  |  |  |  |
| 2. TraumaExp | -.385 | 1.000 |  |  |  |  |  |  |  |  |  |  |  |
| 3. TraumaSeq | .235 | -.455 | 1.000 |  |  |  |  |  |  |  |  |  |  |
| 4. Stress | -.427 | .295 | -.154 | 1.000 |  |  |  |  |  |  |  |  |  |
| 5. Anx | -.447 | .316 | -.146 | .899 | 1.000 |  |  |  |  |  |  |  |  |
| 6. Depr | -.464 | .285 | -.141 | .870 | .849 | 1.000 |  |  |  |  |  |  |  |
| 7. Resil | .315 | -.159 | .056 | -.647 | -.632 | -.669 | 1.000 |  |  |  |  |  |  |
| 8. Som | -.407 | .254 | -.166 | .708 | .738 | .687 | -.567 | 1.000 |  |  |  |  |  |
| 9. Eat | -.186 | .221 | -.118 | .368 | .339 | .330 | -.256 | .306 | 1.000 |  |  |  |  |
| 10. SoC | -.307 | .265 | -.100 | .560 | .621 | .571 | -.486 | .464 | .308 | 1.000 |  |  |  |
| 11. ReApp | -.033 | .144 | -.023 | .024 | .053 | -.042 | .111 | .004 | .099 | .073 | 1.000 |  |  |
| 12. Supp | -.152 | .070 | -.034 | .150 | .166 | .268 | -.170 | .165 | .077 | .168 | .233 | 1.000 |  |
| 13. Social | -.419 | .358 | -.145 | .743 | .740 | .736 | -.640 | .600 | .432 | .628 | .098 | .268 | 1.000 |

**Note.** Values represent latent factor covariances estimated from a confirmatory factor analysis regarding the final model of the clinical and psychosocial factors cluster. Latent Factors: Ctrauma: Childhood Trauma; TraumaExp: Trauma Exposure; TraumaSeq: Trauma Sequelae; Stress: Stress; ANX: Anxiety; Depr: Depression; Resil: Resilience; Som: Somatization; Eat: Eating Behavior; SoC: Sense of Control; ReApp: Reappraissal; Suppr: Suppression; Social: Social Support.

Förstner, B.R., Böttger, S.J., Seegert, T., Kerber, A., Terhorst, Y., Heinz, A., Dziobek, I., Voigt, B., Schneider, S., Opel, N., Walter, M., Braun, U., Meyer-Lindenberg, A., Koutsouleris, N., Falkai, P., Schmitz, N., Fallgatter, A.J., Sünkel, U., Lipinski, S., Rapp, M.A., Tschorn, M. + MDS Expert Group -

Psychometric validation of Research Domain Criteria, General Health, and Clinical-Psychosocial parts of the Minimum Dataset of the German Center for Mental Health - Informing a Delphi Process

European Archives of Psychiatry and Clinical Neuroscience

Corresponding Author: Bernd R Förstner bernd.foerstner@uni-potsdam.de
